# An optimized, rhamnolipid-containing cell-free filtrate from *Pseudomonas aeruginosa* 8–7 exhibits broad-spectrum antifungal activity and exceptional environmental stability

**DOI:** 10.3389/fpls.2026.1809669

**Published:** 2026-06-10

**Authors:** Li-ming Dai, Li-li He, Yue-ye Deng, Lan-lan Li, Yi-xian Liu, Yu-ping Shi, Zhi-ying Cai

**Affiliations:** Tropical Crop Diseases Research Group, Research Center of Plant Protection, Yunnan Key Laboratory of Sustainable Utilization Research on Rubber Tree, Yunnan Institute of Tropical Crops, Jinghong, Yunnan, China

**Keywords:** antifungal activity, cell-free filtrate, fermentation optimization, plant pathogens, *Pseudomonas aeruginosa* 8-7, rhamnolipids, stability

## Abstract

**Introduction:**

The biocontrol strain *Pseudomonas aeruginosa* 8-7 (PA8-7) produces antimicrobial metabolites, yet its application is limited by low yield and insufficiently characterized environmental stability.

**Methods:**

Fermentation was optimized through medium screening, single-factor experiments, and orthogonal array design. The resulting cell‑free filtrate (CFF) was evaluated for antifungal activity against 19 tropical crop pathogens, environmental stability (thermal, pH, UV, reductants, metal ions), rhamnolipid composition (TLC, HPLC‑MS, ¹H NMR), field efficacy against rubber tree powdery mildew (*Oidium heveae*), and biosafety on soybean seedlings.

**Results:**

The optimal medium (15 g/L acid‑hydrolyzed casein, 25 mL/L glycerol, 1 g/L MgSO₄·7H₂O, 1.5 g/L K₂HPO₄, pH 6.5) and culture conditions (50 mL/500 mL flask, 220 rpm, 10% inoculum, 36 °C, 5 days) increased antifungal activity against *Colletotrichum alienum* by 54% to 89.81%. The CFF exhibited broad‑spectrum activity against all 19 pathogens, with the highest inhibition against *Phytophthora litchii* (94.56 ± 1.38%) and *Phytophthora melonis* (93.87 ± 1.69%). It retained >87% activity after autoclaving (121 °C, 30 min), remained stable across pH 1–12, and tolerated UV, reductants and most metal ions. Eighteen rhamnolipid congeners (9 mono‑, 9 di‑) were identified. Field trials showed that a 10‑fold diluted CFF reduced the disease index of rubber tree powdery mildew from 23.55 to 21.11 within 7 days (73.23% control efficacy), with no phytotoxicity on soybean seedlings.

**Conclusion:**

The optimized CFF combines broad‑spectrum activity, exceptional stability, and field efficacy with biosafety, making it a promising low‑cost biocontrol candidate for sustainable tropical agriculture.

## 1 Introduction

Tropical crops, such as rubber trees, litchi, muskmelon, and citrus, are economically vital in China’s tropical regions (Zhu et al., 2022). Natural rubber, in particular, is not only a crucial cash crop but also a strategic industrial material, whose stable supply is essential for national economic security (Ramaer, 1935). However, the warm and humid climate in these regions fosters pathogen proliferation, leading to frequent disease outbreaks (Bebber and Gurr, 2015). For instance, rubber tree anthracnose, caused primarily by *Colletotrichum* species, causes severe defoliation and dieback, delaying latex tapping and reducing yields (Cao et al., 2019). Litchi downy blight, caused by the oomycete *Peronophythora litchii*, can lead to yield losses of 30–80% (Xing et al., 2018). Current management relies heavily on synthetic fungicides, but their long-term use has resulted in environmental pollution, hazardous residues, and pathogen resistance, threatening sustainable production (Carvalho and Fernando, 2017; Hawkins et al., 2018).

Biological control, valued for its environmental compatibility and target specificity, is a promising strategy for managing tropical crop diseases (Khl et al., 2019). Among biocontrol microorganisms, *Pseudomonas aeruginosa* is a well-studied and versatile genus (Gross and Loper, 2009; Weller, 2007). Its efficacy is attributed to a diverse array of antimicrobial secondary metabolites, including siderophores (e.g., pyoverdine), phenazine derivatives, polyketides like pyoluteorin, and rhamnolipid biosurfactants (Biessy and Filion, 2021; Cornelis and Dingemans, 2013; Jiang et al., 2015; Pierson and Pierson, 2010; Raaijmakers and Mazzola, 2012; Yang et al., 2024). Rhamnolipids, anionic biosurfactants synthesized by *P. aeruginosa*, are non-toxic, biodegradable, and can disrupt fungal cell walls and membrane permeability (Kim et al., 2000; Müller et al., 2012; Sekhon Randhawa and Rahman, 2014).

However, the industrialization of biocontrol agents faces two major bottlenecks: fermentation optimization and environmental stability. Fermentation conditions, particularly carbon and nitrogen sources, critically influence the yield of secondary metabolites (Cheng et al., 2024; Li et al., 2024; Liu et al., 2024). Moreover, the stability of these metabolites under field conditions is paramount for practical application. For example, phenazines produced by some *P. aeruginosa* strains are highly sensitive to acidic pH and certain metal ions (Qiao et al., 2015), which limits their use in variable environments. Rhamnolipids are among the most widely studied biosurfactants in recent years (Yao et al., 2021). They are anionic biosurfactants synthesized by *P. aeruginosa* via fermentation, composed of one to two rhamnose molecules as the hydrophilic group and one to two saturated or unsaturated fatty acids as the hydrophobic group (Sonbhadra et al., 2025). They are non-toxic, harmless, and biodegradable, and are mainly used as agricultural adjuvants in agriculture. Rhamnolipids can destroy the cell wall of fungal hyphae, alter cell membrane permeability, and thereby inhibit hyphal growth (Chen et al., 2021).

*Pseudomonas aeruginosa* 8-7 (PA 8-7), isolated from rubber tree rhizosphere soil (CGMCC No. 20694) (Dai et al., 2025), shows potential against tropical crop pathogens. However, its application is hindered by low antimicrobial yield and insufficiently characterized stability under harsh conditions (e.g., high temperature and UV radiation). Several studies have reported antifungal activities of cell-free filtrates (CFFs) from *P. aeruginosa* against plant pathogens, including strains SWUC02, GSE 18/GPS 21, and SNTKU16 (Pringsulaka et al., 2025; Kishore et al., 2005; Thammasittirong et al., 2025). Nevertheless, critical gaps remain: most studies used unoptimized fermentation conditions, lacked systematic stability evaluations under extreme environments, and did not assess biosafety or field efficacy against major tropical diseases.

The present study was therefore designed to address these gaps through comprehensive fermentation optimization, rigorous stability characterization, compositional analysis of active metabolites, and field validation combined with safety evaluation. Specifically, we aimed to (1) optimize the fermentation medium and conditions to enhance antimicrobial activity; (2) characterize the rhamnolipid composition by TLC and HPLC-MS and systematically evaluate the CFF’s broad−spectrum efficacy and environmental stability (thermal, pH, UV, reductant, and metal ion tolerance). Notably, although *P. aeruginosa* is an opportunistic human pathogen, this study exclusively used sterile CFF—not live bacteria—eliminating the risk of infection and ensuring biosafety. Our findings provide a solid foundation for developing PA 8–7 as a promising biocontrol agent for sustainable tropical agriculture.

## 2 Materials and methods

### 2.1 Microbial strains and chemical reagents

Biocontrol strain: PA 8–7 was isolated and purified from the rhizosphere soil of rubber trees by the Plant Protection and Microbial Utilization Research Center, Yunnan Institute of Tropical Crops. It has been deposited in the China General Microbiological Culture Collection Center with the accession number CGMCC No.20694 (Dai et al., 2025). http://epub.cnipa.gov.cn/cred/CN119799573B

Pathogens: A total of 19 species of tropical crop pathogens were used in the test, all preserved by the Tropical Crop Disease Research Group of Yunnan Institute of Tropical Crops. These pathogens include *Colletotrichum gloeosporioides*, *Corynespora cassiicola*, *Colletotrichum alienum, Colletotrichum siamense*, *Alternaria heveae*, *Fusarium solani*, *Pestalotiopsis microspora*, *Calonectria pentaseptata*, *Peronophythora litchii*, *Phytophthora melonis Katsura*, *Phytophthora vignae Purss*, *Fusarium oxysporum* f. sp. cubense, *Phytophthora capsici*, *Rhizopus stolonifer*, *Alternaria solani*, *Botrytis cinerea*, *Alternaria nicotiana*, *Alternaria alternata*, and *Diaporthe passifloricola*.

Reagents: Analytical grade reagents including anhydrous ethanol, yeast extract, tryptone, NaCl, K_2_HPO_4_, KH_2_PO_4_, MgSO_4_·7H_2_O, potassium nitrate, sucrose, peptone, beef extract, soluble starch, glycerol, CuSO_4_, FeCl_3_, NiSO_4_, MgCl_2_, KCl, ZnSO_4_, CaCl_2_, BaCl_2_, and Pb(NO_3_)_2_ were purchased from Sangon Biotech Co., Ltd. Casein acid hydrolysate (C8221), Glucose, maltose, lactose, galactose, fructose, and EDTA-Na_2_ were purchased from Solarbio Science & Technology Co., Ltd., Beijing. Casein acid hydrolysate (C822594) was purchased from Macklin Biochemical Co., Ltd., Shanghai. Mineral oil, soybean oil, corn oil, peanut oil, rapeseed oil, olive oil, palm oil, Xylose, and mannitol were purchased from Yuanye Bio-Technology Co., Ltd. Soybean meal and corn steep liquor Beijing Hongrun Baoshun Technology Co., Ltd. All chemical reagents used in this study were of analytical grade. Detailed information regarding the reagents, including manufacturer names and catalog numbers, is provided in **Supplementary Table 1**. All experiments involving live PA8–7 were conducted under biosafety level 2 (BSL-2) containment. For antifungal activity assays, the CFF was prepared by filtration through a 0.22 μm membrane to remove bacterial cells. For thermal stability tests and field trials, the CFF was sterilized by autoclaving (121 °C, 30 min) to completely inactivate viable cells.

### 2.2 Medium preparation

### 2.3 Preparation of fermentation seed culture

PA 8–7 was revived on LB agar at 37 °C for 24 h. A single colony was inoculated into 100 mL of LB broth in a 300 mL flask and incubated at 36 °C with shaking at 180 rpm for 12 h until the culture reached the exponential phase (OD_600_ = 1.0).

### 2.4 Screening of optimal basal medium for antimicrobial substance production

#### 2.4.1 Screening of media for antimicrobial substance production

Each of the eight candidate fermentation media (100 mL) (**Table 1**) was dispensed into a 500 mL Erlenmeyer flask. The pre-cultured seed culture was inoculated into each flask at an inoculum size of 15% (v/v), followed by incubation at 30 °C with shaking at 180 rpm in the dark for 4 days. The fermentation broth was then centrifuged at 5,860 ×g for 4 min using a HITACHI CT15RE centrifuge (Hitachi Koki Co., Ltd., Japan). The supernatant was collected, transferred into a sterile centrifuge tube, and filtered through a 0.22 µm microporous membrane to obtain the CFFs of the eight candidate fermentation media.

**Table 1:** Composition and rationale for the selection of basal media used for screening antimicrobial substance production by PA 8-7.

Medium	Formula (per 1000 mL)	Selection rationale
Soybean Meal Medium	• Soybean meal: 65 g • Corn steep liquor: 16 g • Glucose: 12 g • Anhydrous ethanol: 22 mL	Promotes pigment and secondary metabolite production in *P. aeruginosa* (Zhou et al., 2015)
Pigment-promoting Medium	• Tryptone: 22 g • Glucose: 20 g • Potassium nitrate: 5 g • pH 7.5	Enhances antimicrobial pigment biosynthesis (Zhou et al., 2015)
Beef Extract-Peptone Medium	• NaCl: 5 g • Peptone: 10 g • Beef extract: 3 g • pH: 7.2–7.4	Classical complex medium for general *Pseudomonas* cultivation (Qiao et al., 2015)
Sucrose Medium	• Sucrose: 3 g • NaCl: 2.5 g • Peptone: 10 g • pH: 7.5	Supports metabolite production in *Pseudomonads* (Qiao et al., 2015)
Terrific Broth (TB) Medium	• Tryptone: 12 g • Yeast extract: 24 g • K_2_HPO_4_: 9.4 g • KH_2_PO_4_: 2.2 g • Glycerol: 4 mL	High-density culture medium for enhanced secondary metabolism (Deng, 2024)
Terrific Broth (FB) Medium	• MgSO_4_·7H_2_O: 1 g • K_2_HPO_4_: 2.5 g • Casein acid hydrolysate: 5 g • Glycerol: 8 mL • pH: 6.5	Specifically formulated for rhamnolipid production in *Pseudomonas* (Li et al., 2024)
Lysogeny Broth (LB)	• Tryptone: 10 g • Yeast extract: 5 g • NaCl: 10 g, • Agar: 15 g	Standard medium for bacterial activation and routine culture (Dai et al., 2022)
Potato Dextrose Agar (PDA)	• Potato: 200 g • Glucose: 20 g • Agar: 15–18 g	Standard medium for fungal pathogen maintenance and antifungal assays (Dai et al., 2022)

#### 2.4.2 Determination of antifungal activity

The mycelial growth rate method was employed using *C. alienum* as the indicator pathogen. *C. alienum* was first activated on PDA plates at 28 °C for 4 days. Mycelial plugs (5 mm in diameter) were then obtained using a sterile cork borer and inoculated onto the center of PDA plates (60 mm diameter) that had been pre-coated with 100 μL of CFF. Each treatment was performed in triplicate. The plates were incubated at 28 °C for 4 days, after which the colony diameter of each plate was measured using the using the cross-bracketing method. The mycelial growth inhibition rate was subsequently calculated.


$$\text{Mycelial growth inhibition }\%=\frac{\text{C}-\text{T}}{\text{C}}\times 100$$

Where C and T are the average diameter (mm) of fungal mycelia in the control and treatment, respectively.

### 2.5 Fermentation formula optimization

The FB medium (best initial activity) was selected for optimization.

#### 2.5.1 Carbon source screening

The FB medium as the basal fermentation medium, glycerol, mineral oil, soybean oil, corn oil, peanut oil, rapeseed oil, olive oil, palm oil, sucrose, xylose, mannitol, glucose, soluble starch, maltose, lactose, galactose, and fructose were separately used as carbon sources at a ratio of 1% (w/v) to replace 8 mL of glycerol for further optimization experiments. The mycelial growth rate method was adopted, with *C. alienum* as the target pathogen, to determine the antifungal activity of 100 μL of CFF from each treatment group, with three replicates per treatment.

#### 2.5.2 Nitrogen source screening

The FB medium as the basal fermentation medium and glycerol as the carbon source, casein acid hydrolysate (Solarbio C8221), casein acid hydrolysate (Macklin C822594), casein hydrolysate, beef extract, tryptone, and yeast extract were separately used as nitrogen sources for further optimization experiments.

#### 2.5.3 Orthogonal experiment optimization

Based on the results of carbon source and nitrogen source screening, four factors including casein acid hydrolysate (Macklin C822594), K_2_HPO_4_, MgSO_4_·7H_2_O and glycerol were selected to conduct a four-factor and four-level orthogonal experiment (**Table 2**). The mycelial growth rate method was adopted, with *C. alienum* as the target pathogen, to determine the antimicrobial activity of 100 μL of CFF from each treatment group, with three replicates per treatment.

**Table 2:** Factors and levels design of orthogonal experiment for fermentation condition optimization.

Level	A Acid-hydrolyzed casein(g/L)	B Glycerol(ml/L)	C K_2_HPO_4_(g/L)	D MgSO_4_·7H_2_O(g/L)
1	5	15	1.5	0.5
2	10	20	2	1
3	15	25	2.5	1.5
4	20	30	3	2

### 2.6 Fermentation condition optimization

Based on the determination of the optimal ratio of components in the basal fermentation medium, the antimicrobial activity of the CFF of the strain against *C. alienum* under different fermentation conditions was determined to identify the optimal fermentation conditions (Liu et al., 2020).

Inoculum size: The fermentation seed culture was inoculated into the optimized fermentation medium at varying inoculum sizes of 5%, 10%, 15%, 20% and 25% (v/v), respectively. The cultures were incubated at 36 °C with shaking at 180 rpm in the dark for 4 days. The optimal inoculum size was then determined based on the antifungal activity of the CFFs.

Culture temperature: On the basis of the optimal inoculum size and using the medium with optimal ratio, the shaker speed was fixed at 180 r/min and the incubation time at 4 days. The shaker incubation temperatures were set at 30 °C, 33 °C, 36 °C and 39°C. The optimal culture temperature was determined by assaying the antimicrobial activity of the CFF.

Shaker speed: Based on the optimal inoculum size and optimal culture temperature, the shaker speeds were set at 120, 140, 160, 180, 200 and 220 r/min to determine the optimal shaker speed.

Incubation time: Under the conditions of optimal inoculum size, optimal culture temperature and optimal shaker speed, the cultures were incubated for 4, 5, 6, 7, and 8 days, respectively, to determine the optimal incubation time.

Medium volume per flask: Keeping other culture conditions unchanged, 50, 100, 150, and 200 mL of the medium were dispensed into 500 mL Erlenmeyer flasks, respectively, to determine the optimal medium volume per flask.

During the optimization of the above conditions, in addition to determining the antifungal inhibition rate of the sterile fermentation broth, the rhamnolipid content was simultaneously quantified via OD_625_ measurement. Detailed methods are described in Supplementary Methods.

### 2.7 Determination of antifungal spectrum of fermentation filtrate from optimized medium

To evaluate the inhibitory spectrum, the CFF obtained under optimized conditions was tested against 19 tropical crop pathogens using the mycelial growth rate method. Each plate received 100 μL of CFF, and three replicates were included per pathogen. The inhibition rate was calculated to assess the antifungal activity against various pathogens causing tropical crop diseases.

### 2.8 Determination of CFF stability

#### 2.8.1 Light stability

To evaluate the photostability of the CFF, two independent exposure experiments were conducted under natural light and ultraviolet (UV) irradiation, respectively (Liu et al., 2020 and Qiao et al., 2015). For natural light treatment, the CFF was exposed to ambient light for 1, 2, 3, 4, and 5 days, with samples collected daily for antimicrobial activity assessment. For UV treatment, aliquots of the CFF were placed 15 cm away from a 30 W UV lamp (UV-C) and irradiated for 30, 60, 90 and 120 min. Untreated CFF served as the control. All treatments were performed in triplicate.

Following exposure, mycelial plugs (5 mm in diameter) of *C. alienum* were inoculated onto PDA plates supplemented with 100 μL of CFF from each experimental group. The plates were incubated upside down at 28 °C for 4 days. The colony diameter of each plate was measured using the cross-bracketing method, and the mycelial growth inhibition rate was calculated to determine the effect of light exposure on the antifungal activity of the CFF.

#### 2.8.2 Temperature stability

To assess the thermal stability of the CFF, three heat treatment regimens were applied (Liu et al., 2020 and Qiao et al., 2015). Aliquots of the CFF were subjected to (i) incubation in a water bath at 80 °C for 1 h, (ii) incubation in a water bath at 100 °C for 1 h, and (iii) autoclaving at 121 °C for 30 min. After cooling to room temperature, 100 μL of each treated CFF sample was evenly spread onto PDA plates. Mycelial plugs (5 mm in diameter) of *C. alienum* were then inoculated onto the center of the plates. The plates were incubated upside down at 28 °C for 4 days. Untreated CFF served as the control. All treatments were performed in triplicate. Following incubation, the colony diameter of each plate was measured, and the mycelial growth inhibition rate was calculated to evaluate the effect of temperature on the antifungal activity of the CFF.

#### 2.8.3 pH stability

To evaluate the pH stability of the CFF, the pH of the CFF was adjusted to 1, 3, 4, 5, 7, 9, 11, and 12 using 0.1 mol/L HCl or 0.1 mol/L NaOH, respectively (Liu et al., 2020 and Qiao et al., 2015). After standing at room temperature for 30 min, the pH of each sample was readjusted to its original value (pH 7.2). An aliquot of 100 μL from each pH-treated CFF sample was then spread onto PDA plates. Mycelial plugs (5 mm in diameter) of *C. alienum* were inoculated onto the center of the plates. The plates were incubated upside down at 28 °C for 4 days. Untreated CFF served as the control. All treatments were performed in triplicate. Following incubation, the colony diameter of each plate was measured, and the mycelial growth inhibition rate was calculated to assess the effect of pH on the antifungal activity of the CFF.

#### 2.8.4 Reductant stability

To evaluate the stability of the CFF under reducing conditions, the CFF was supplemented with sodium sulfite (Na_2_SO_3_) at final concentrations of 0.4×10^-4^, 5×10^-4^, 2×10^-^³, and 5×10^-^³ mol/L, respectively (Liu et al., 2020 and Qiao et al., 2015). The mixtures were allowed to stand at room temperature for 30 min. Subsequently, 100 μL of each treated CFF sample was spread onto PDA plates. Mycelial plugs (5 mm in diameter) of *C. alienum* were inoculated onto the center of the plates. The plates were incubated upside down at 28 °C for 4 days. After incubation, the colony diameter of each plate was measured, and the mycelial growth inhibition rate was calculated. To exclude the direct effect of Na_2_SO_3_ on pathogen growth, Na_2_SO_3_ solutions at the corresponding concentrations without CFF were used as additional controls, and the colony diameters of *C. alienum* in these control groups were also determined.

#### 2.8.5 Metal ions stability

To evaluate the stability of the CFF in the presence of metal ions, stock solutions (0.1 mol/L) of various CuSO_4_, EDTA-Na_2_, FeCl_3_, NiSO_4_, NaCl, MgCl_2_, KCl, ZnSO_4_, CaCl_2_, BaCl_2_, and Pb (NO_3_) _2_ were freshly prepared (Qiao et al., 2015). Aliquots of 10 μL from each salt solution were transferred into separate 1.5 mL microcentrifuge tubes, followed by the addition of 190 μL of CFF to achieve a final metal ion concentration of 0.005 mol/L. The mixtures were allowed to stand at room temperature for 5 h. Subsequently, 100 μL of each treated CFF sample was spread onto PDA plates. Mycelial plugs (5 mm in diameter) of *C. alienum* were inoculated onto the center of the plates. The plates were incubated upside down at 28 °C for 4 days. After incubation, the colony diameter of each plate was measured, and the mycelial growth inhibition rate was calculated. To exclude the direct effect of metal ions on pathogen growth, salt solutions at the corresponding concentrations without CFF were used as additional controls, and the colony diameters of *C. alienum* in these control groups were also determined.

### 2.9 Extraction and determination of rhamnolipids

Rhamnolipids were extracted from the CFF using the acid precipitation method (Shuang et al., 2017). Briefly, the pH of the CFF was adjusted to 2.0 using 6 mol/L HCl and stored at 4 °C overnight to precipitate the rhamnolipids. The precipitate was collected by centrifugation at 10,000 × g for 20 min at 4 °C and then extracted three times with ethyl acetate. The organic phase was pooled and evaporated to dryness under reduced pressure at 40 °C using a rotary evaporator. The obtained crude extract was dissolved and filtered through a 0.22 µm membrane prior to analysis.

Rhamnolipid quantification was performed using the anthrone-sulfuric acid method (Qi and Xu, 2010). Briefly, L-rhamnose standard solutions (0–100 mg/L) were prepared and reacted with freshly prepared anthrone reagent (0.2% in concentrated sulfuric acid) at 100 °C for 15 min. After cooling, the absorbance at 625 nm was measured to construct a standard curve. Rhamnolipid concentration in the samples, expressed as rhamnose equivalents, was then calculated based on this curve. This method is widely used for preliminary screening and comparative purposes in fermentation optimization studies (Wei et al., 2005; Lotfabad et al., 2010). However, we acknowledge that it may overestimate rhamnolipid concentrations due to potential cross-reactivity with other carbohydrate-containing compounds in the complex fermentation broth. Therefore, the reported value should be interpreted as an estimate of rhamnolipid content in rhamnose equivalents.

The composition of the extracted rhamnolipids was analyzed by TLC, HPLC-MS, and ¹H NMR spectroscopy. Detailed procedures for these analyses are provided in the Supplementary Methods (Bai et al., 2012).

### 2.10 Safety evaluation of the sterile CFF on soybean seedlings

To assess the biosafety of the autoclaved CFF for agricultural application, a pot experiment was conducted using 15−day−old soybean seedlings (cultivar ‘Jiefeng Chaochang 98’). Seeds were germinated and grown under uniform conditions. Healthy seedlings of similar size were selected and divided into three groups (15 seedlings per group, three biological replicates): Group A (control): foliar sprayed with sterile un-inoculated FA medium; Group B (diluted): foliar sprayed with 10−fold diluted autoclaved CFF; Group C (undiluted): foliar sprayed with undiluted autoclaved CFF. All seedlings were maintained under identical conditions (light, temperature, watering) and observed daily for 7 days. Phytotoxicity symptoms, including leaf color changes, curling, deformity, chlorosis, necrosis, and wilting, were recorded.

### 2.11 Field efficacy of the CFF against rubber tree powdery mildew (*Oidium heveae*)

The trial was conducted at the rubber tree germplasm nursery of the Yunnan Institute of Tropical Crops using the susceptible clone ‘GT1’ at the bronze leaf stage. Two treatments were applied: (i) 10−fold diluted autoclaved CFF, and (ii) sterile un−inoculated FA medium as control. Each treatment was applied to 10 trees.

Before treatment and 7 days after treatment, 10 leaves per tree (100 leaves total per treatment) were randomly collected and assessed for disease severity according to the Chinese agricultural standard NY/T 1089−2015 (Ministry of Agriculture, 2015). Disease index (DI) and control efficacy (CE) were calculated as follows:


$$DI=\frac{\sum \left(ni\times i\right)}{N\times 9}\times 100$$

Where ni is the number of leaves in each disease grade (0–9), i is the grade number, and N is the total number of leaves.


$$CE=[1-\frac{DI_{control,\;before}\times DI_{treatemnt,after}}{DI_{control,after}\times DI_{treatment,\;before}}]\times 100\%$$

All treatments were performed in triplicate, and data are presented as mean ± SD.

### 2.12 Data processing

Data were analyzed using SPSS Statistics 20. Three independent biological replicates were performed, each with three technical replicates. Results are expressed as mean ± standard deviation (SD). Significant differences (*P* < 0.05) were determined by one-way analysis of variance (ANOVA) followed by Duncan’s new multiple range test.

## 3 Results

### 3.1 Fermentation medium screening

**Figure 1:**
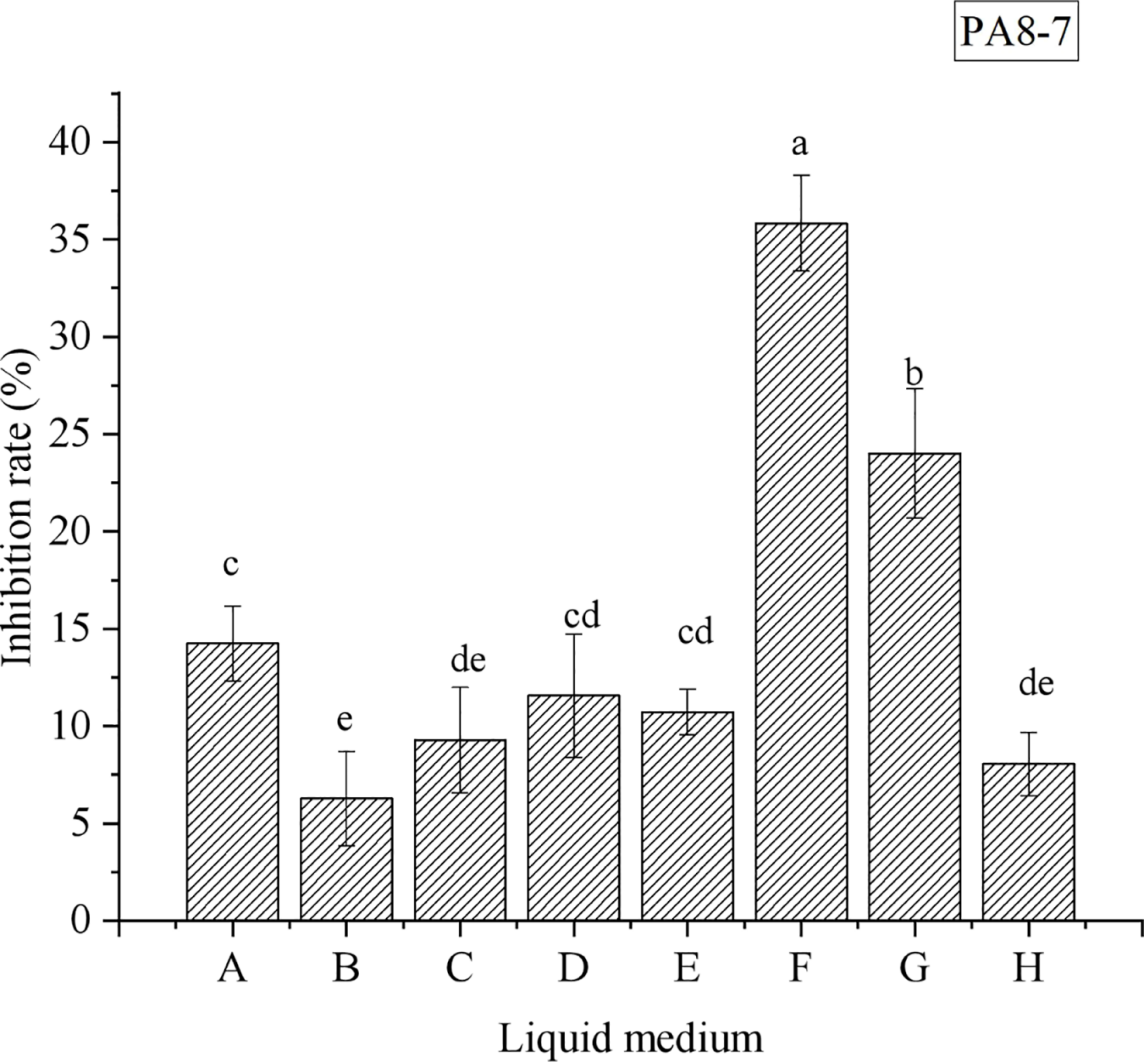
Antifungal activity of PA 8–7 sterile fermentation filtrates cultured in different screening media against *C. alienum.* Representative images: **(A)** PDA; **(B)** sucrose; **(C)** LB; **(D)** soybean meal; **(E)** pigment-promoting; **(F)** FB; **(G)** TB; **(H)** beef extract-peptone. All the data are expressed as means ± SD of three independent experiments. Statistical analysis was performed using one-way ANOVA at *p* < 0.05 designated by superscripts a, b, c, d, and e.

There were significant differences in the antifungal rates of CFFs prepared from 8 media against *C. alienum* of rubber trees (**Figure 1**). The FB medium yielded the highest inhibition rate against *C. alienum* (35.83% ± 2.46%), which was significantly greater than those of the other media (e.g., TB medium: 24.02% ± 3.33%). Therefore, the FB medium was selected for further optimization.

### 3.2 Fermentation formula optimization

#### 3.2.1 Carbon sources and nitrogen sources

Carbon Source: CFFs of PA 8–7 were obtained using 17 carbon sources, among which the filtrates from 9 carbon sources exhibited certain inhibitory effects against *C. alienum* (**Table 3**). When glycerol was used as the carbon source, the fermentation broth of the strain showed the highest antimicrobial rate, reaching 42.38% ± 2.7%. The CFFs also presented antimicrobial activity when soybean oil, corn oil, peanut oil, rapeseed oil, olive oil, and palm oil were used as carbon sources.

**Table 3:** Effects of carbon source media on antifungal activity of PA 8–7 sterile fermentation filtrates.

Carbon sources	Inhibition rate(%)
Glycerol	42.38 ± 2.7^a^
Mannitol	41.16 ± 2.49^ab^
Rapeseed oil	39.35 ± 4.11^abc^
Peanut oil	38.52 ± 3.96^bc^
Olive oil	38.39 ± 1.82^bc^
Corn oil	37.84 ± 1.45^bc^
Palm oil	37.54 ± 2.12^bc^
Soybean oil	36.26 ± 1.88^c^
Fructose	5.24 ± 1.27^d^
Sucrose	0
Mineral oil	0
Xylose	0
Galactose	0
Soluble starch	0
Lactose	0
Maltose	0
Glucose	0

Statistical analysis was performed using one-way ANOVA at *p* < 0.05 designated by superscripts a, b, c, and d.

No antimicrobial activity was detected in the CFFs of the strain when mineral oil, xylose, sucrose, galactose, soluble starch, lactose, glucose, and maltose served as carbon sources. The CFF showed extremely low antimicrobial rate when fructose was used as the carbon source. The antimicrobial rate of the strain fermentation broth was comparable between mannitol and glycerol as carbon sources. Considering the cost factor, glycerol was selected as the optimal carbon source for the liquid fermentation medium.

Nitrogen Source: CFFs of PA 8–7 were obtained using 6 nitrogen sources, all of which exhibited inhibitory effects against *C. alienum* (**Table 4**). Among these nitrogen sources, casein acid hydrolysate (C822594) and casein hydrolysate yielded the highest antimicrobial rates, with values of 62.96% ± 0.98% and 60.85% ± 2.49%, respectively. Tryptone and yeast extract ranked next, showing antimicrobial rates of 56.57% ± 2.77% and 54.45% ± 1.23%, respectively. Beef extract showed the lowest antimicrobial rate at 37.27% ± 2.60%. Therefore, casein acid hydrolysate (C822594) was selected as the optimal nitrogen source for the liquid fermentation medium.

**Table 4:** Effects of different nitrogen source media on antifungal activity of PA 8–7 sterile fermentation filtrate.

Nitrogen source	Inhibition rate(%)
Casein acid hydrolysate C822594	62.96 ± 0.98^a^
Casein hydrolysate yielded	60.85 ± 2.49^a^
Tryptone	56.57 ± 2.77^b^
Yeast powder	54.45 ± 1.23^b^
Casein acid hydrolysate C8221	50.25 ± 2.71^c^
Beef extract	37.27 ± 2.60^d^

Statistical analysis was performed using one-way ANOVA at *p* < 0.05 designated by superscripts a, b, c, and d.

#### 3.2.2 Orthogonal experiment

Based on the results of carbon source and nitrogen source screening, four factors including casein acid hydrolysate (C822594), K_2_HPO_4_, MgSO_4_·7H_2_O and glycerol were selected to conduct a four-factor and four-level orthogonal experiment. The results of the orthogonal experiment presented in **Table 5** showed that the order of influence of the four factors on the inhibition rate against *C. alienum* was casein acid hydrolysate > glycerol > K_2_HPO_4_ > MgSO_4_·7H_2_O. The optimal level combination was A_3_B_3_C_1_D_2_, namely combination No. 11. Among the 16 groups in the analysis of variance (ANOVA) of antimicrobial effects, combination No. 11 exhibited the highest inhibition rate against *C. alienum*. The optimized FB fermentation medium was designated as FA fermentation medium, with the components as follows: casein acid hydrolysate (C822594) 15 g/L, glycerol 25 mL/L, MgSO_4_·7H_2_O 1 g/L, K_2_HPO_4_ 1.5 g/L, and pH 6.5.

**Table 5:** Orthogonal array design L_16_(4^4^) and antifungal activity results for optimization of fermentation medium components.

Number	Factor				Inhibition rate%
	A	B	C	D	
1	1	1	1	1	63.67 ± 3.43
2	1	2	2	2	60.96 ± 2.50
3	1	3	3	3	62.92 ± 3.43
4	1	4	4	4	60.88 ± 1.74
5	2	1	2	3	66.02 ± 1.92
6	2	2	1	4	64.21 ± 1.82
7	2	3	4	1	65.39 ± 3.86
8	2	4	3	2	64.01 ± 1.89
9	3	1	3	4	61.96 ± 3.16
10	3	2	4	3	65.94 ± 2.47
11	3	3	1	2	70.37 ± 2.19
12	3	4	2	1	65.29 ± 1.75
13	4	1	4	2	64.78 ± 2.26
14	4	2	3	1	64.45 ± 1.47
15	4	3	2	4	65.94 ± 2.07
16	4	4	1	3	63.83 ± 2.47
K1	62.11	64.11	65.52	64.70	
K2	64.91	63.89	64.55	65.03	
K3	65.89	66.16	63.34	64.68	
K4	64.75	63.50	64.25	63.25	
R	3.78	2.65	2.18	1.78	

A, casein acid hydrolysate (C822594)(g/L); B, K_2_HPO_4_ (g/L); C, MgSO_4_·7H_2_O (g/L); D, glycerol (mL/L). K1-K4, mean inhibition rate at each level; R, range (maximum - minimum). Values are mean ± SD (n=3).

### 3.3 Fermentation condition optimization

#### 3.3.1 Inoculum size

**Figure 2:**
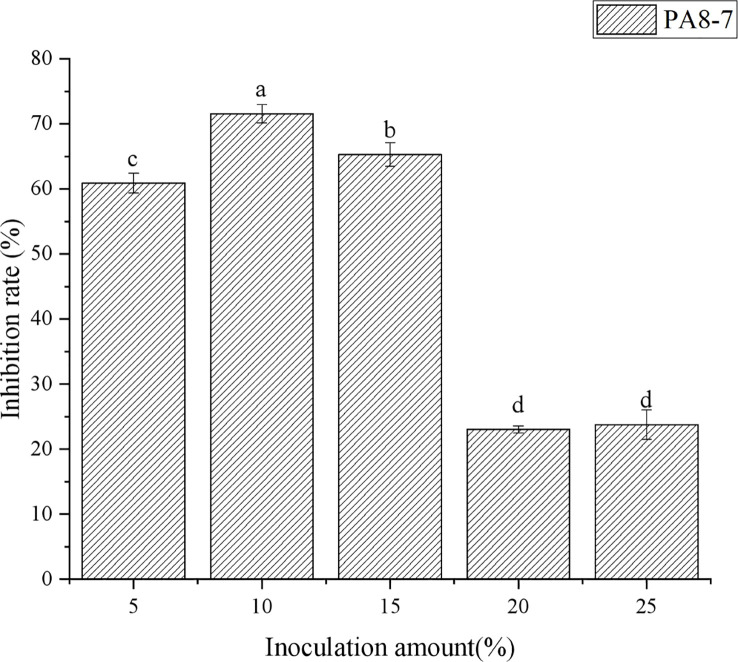
Effects of different inoculum sizes on antifungal activity of PA 8–7 sterile fermentation filtrates. All the data are expressed as means ± SD of three independent experiments. Statistical analysis was performed using one-way ANOVA at *p* < 0.05 designated by superscripts a, b, c, and d.

The antimicrobial rate peaked at 71.56% ± 1.44% when the inoculum size was 10%. When the inoculum size was lower than 10%, the antimicrobial rate increased with the rise of inoculum size (the rate was only 60.88% ± 1.53% at an inoculum size of 5%). When the inoculum size was higher than 10%, the antimicrobial rate decreased with the increase of inoculum size (the rate dropped to 23.77% ± 2.25% at an inoculum size of 25%) (**Figure 2**). Therefore, 10% was selected as the optimal inoculum size.

#### 3.3.2 Culture temperature

**Figure 3:**
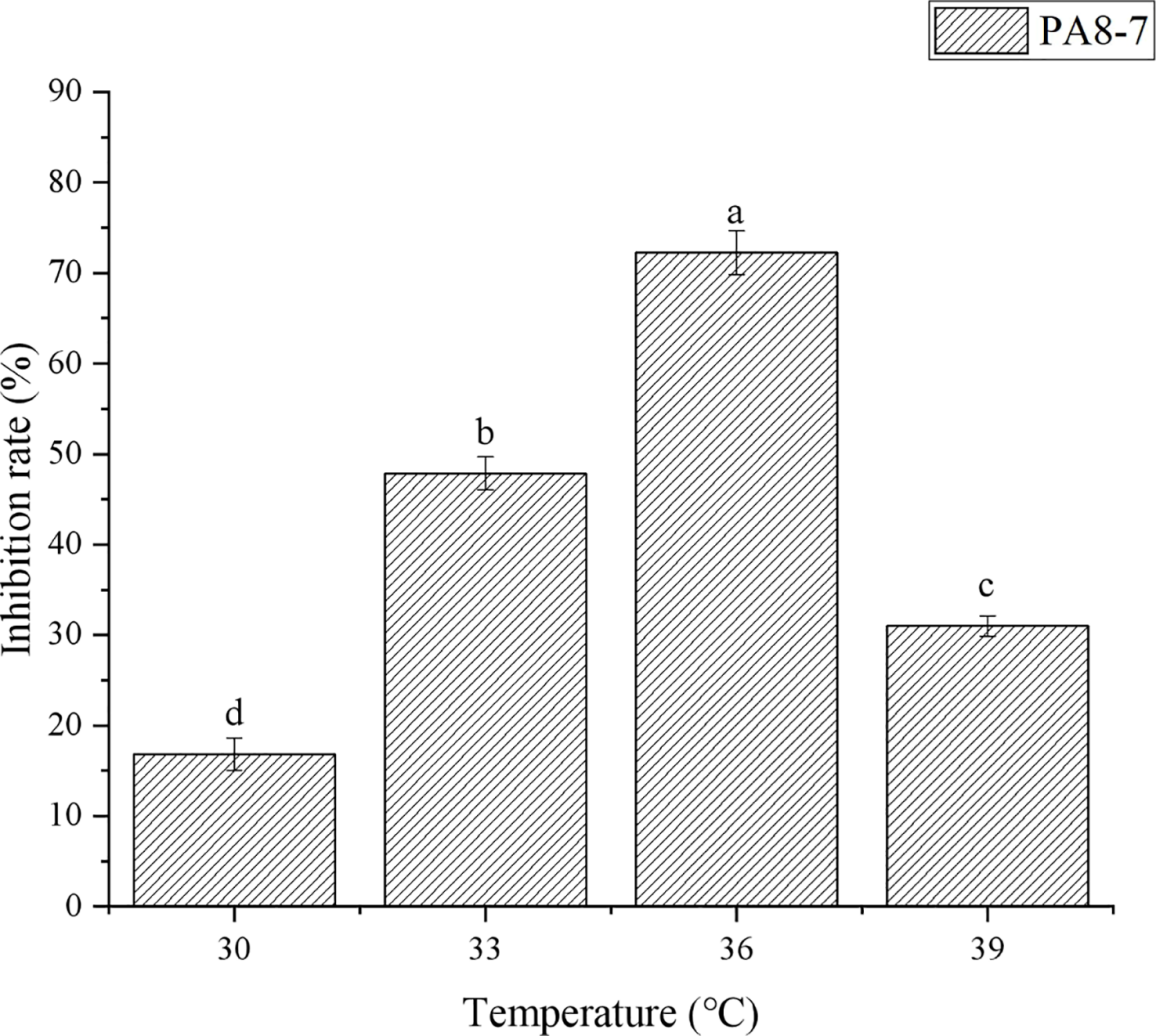
Effects of different culture temperatures on antifungal activity of PA 8–7 sterile fermentation filtrates. All the data are expressed as means ± SD of three independent experiments. Statistical analysis was performed using one-way ANOVA at *p* < 0.05 designated by superscripts a, b, c, and d.

The highest antimicrobial rate of 72.24% ± 2.41% was achieved at a culture temperature of 36 °C. The antimicrobial rates at 33 °C and 39 °C were relatively lower, reaching 47.85% ± 1.83% and 31% ± 1.13%, respectively (**Figure 3**). Therefore, 36 °C was selected as the optimal culture temperature.

#### 3.3.3 Shaker speed

**Figure 4:**
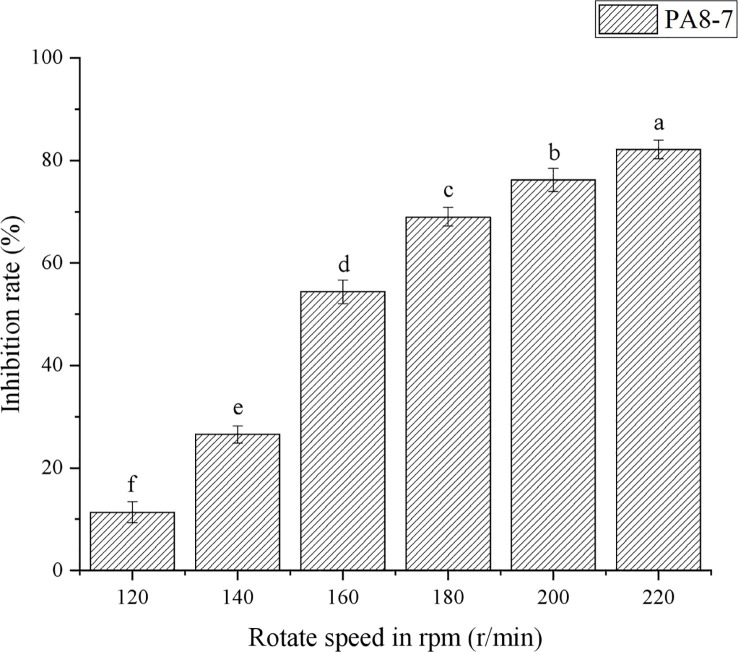
Effects of different culture rotation speeds on antifungal activity of PA 8–7 sterile fermentation filtrates. All the data are expressed as means ± SD of three independent experiments. Statistical analysis was performed using one-way ANOVA at *p* < 0.05 designated by superscripts a, b, c, d, e, and f.

The antimicrobial rate reached 81.45% ± 1.83% at a shaker speed of 220 r/min, which was significantly higher than that at other speeds (*P* < 0.05). When the speed was lower than 220 r/min, the antimicrobial rate increased with the rise of shaker speed, with the rate only being 11.38% ± 2.05% at 120 r/min (**Figure 4**). Therefore, 220 r/min was selected as the optimal shaker speed.

#### 3.3.4 Incubation time

**Figure 5:**
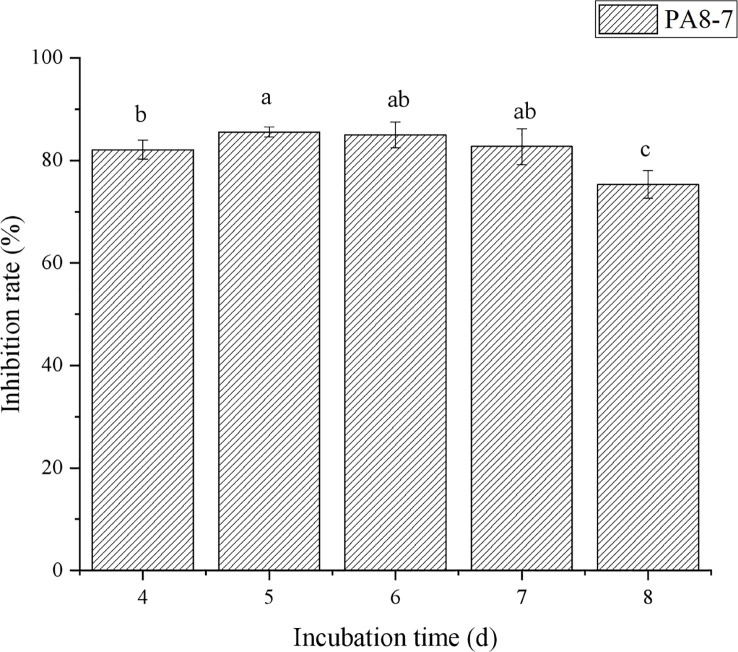
Effects of different culture times on antifungal activity of PA 8–7 sterile fermentation filtrates. All the data are expressed as means ± SD of three independent experiments. Statistical analysis was performed using one-way ANOVA at *p* < 0.05 designated by superscripts a, b, and c.

Among the tested incubation durations, the highest antimicrobial activity was achieved at 5 days, with an inhibition rate of 85.56% ± 1.86%. Comparable rates were observed at 4 days (82.08% ± 1.86%), 6 days (84.96% ± 2.51%), and 7 days (82.71% ± 3.50%) (**Figure 5**). Based on these results, an incubation time of 5 days was selected as optimal for subsequent experiments.

#### 3.3.5 Medium volume per flask

**Figure 6:**
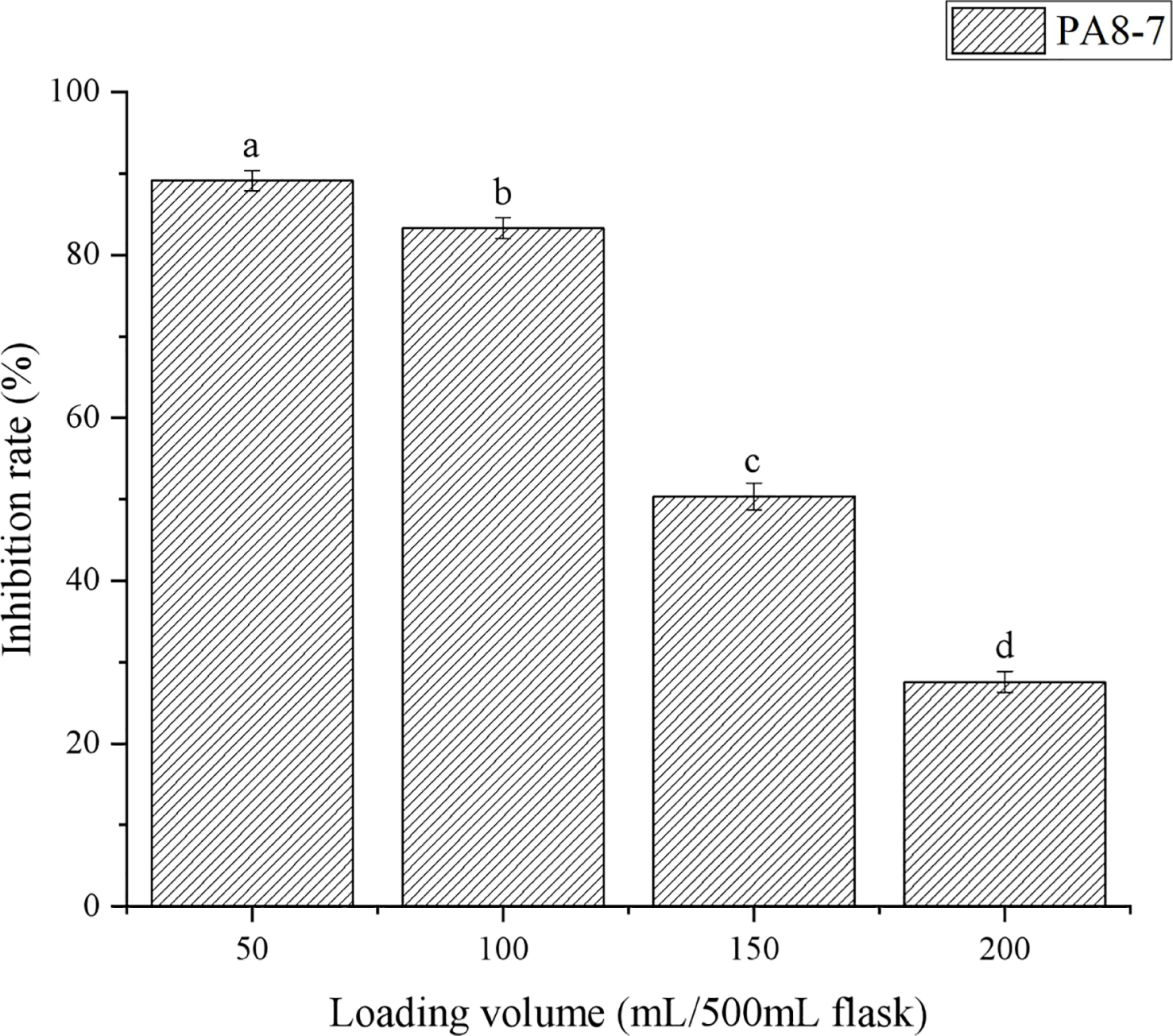
Effects of different liquid loading volumes on antifungal activity of PA 8–7 sterile fermentation filtrates. All the data are expressed as means ± SD of three independent experiments. Statistical analysis was performed using one-way ANOVA at *p* < 0.05 designated by superscripts a, b, c, and d.

The highest antimicrobial rate (89.12% ± 1.24%) was achieved with a medium volume of 50 mL per 500 mL flask. When the medium volume was increased to 100 mL per 500 mL flask, the antimicrobial rate decreased to 83.28% ± 1.28%. When the medium volume exceeded 150 mL per 500 mL flask, the antimicrobial rate dropped below 60% (**Figure 6**). Therefore, 50 mL per 500 mL flask was selected as the optimal medium volume.

Throughout the single−factor optimization experiments (inoculum size, temperature, shaker speed, and medium volume), the changes in rhamnolipid OD_625_ were highly consistent with the trends in antifungal activity against *C. alienum*. This consistency suggests that rhamnolipids are the primary contributors to the antifungal activity of the CFF.

To further investigate the time−course of rhamnolipid accumulation and its relationship with bioactivity, we monitored both parameters under the optimized conditions over 8 days (**Supplementary Table 2**). As shown in **Supplementary Table 2**, rhamnolipid OD_625_ increased progressively from day 4 to day 7, reaching a peak value of 2.42 ± 0.04 at day 7, and then remained stable. Antifungal activity reached ≥82% by day 4, with values of 85.56%, 84.96%, and 82.71% on days 5, 6, and 7, respectively. No significant differences were observed among days 4–7 (one−way ANOVA, *p* > 0.05).

Although rhamnolipid accumulation continued to increase after day 5, the antifungal activity did not improve correspondingly. Considering that longer incubation times would increase production costs without additional biocontrol benefit, day 5 was selected as the optimal incubation time for all subsequent experiments. The strong positive correlation between rhamnolipid OD_625_ and antifungal activity further supports the hypothesis that rhamnolipids are the major bioactive components in the CFF.

In summary, the optimal fermentation parameters for PA 8–7 were established as follows: FA medium, a working volume of 50 mL/500 mL flask, an agitation speed of 220 rpm, an inoculum size of 10% (v/v), a cultivation temperature of 36 °C, and an incubation period of 5 days under dark conditions.

### 3.4 Antimicrobial spectrum determination of CFF

The optimized fermentation filtrate exhibited broad-spectrum antifungal activity, inhibiting all 19 tested tropical crop pathogens, albeit with marked variation in susceptibility among species (**Table 6**). The highest inhibitory effects were observed against *P. melonis Katsura* (isolated from melon) and *P. litchii* (isolated from litchi), with inhibition rates of 93.87% ± 1.69% and 94.56% ± 1.38%, respectively. Strong inhibitory activity was also recorded against *D. passifloricola* (88.93% ± 0.54%) and *C. alienum* (89.81% ± 0.44%). In contrast, eight of the tested pathogens exhibited inhibition rates below 50%, including *C. cassiicola*, *F. solani*, *C. pentaseptata*, *A. solani*, *A. nicotiana*, *P. capsici*, *R. stolonifer*, and *F. oxysporum* f. sp.*cubense.*

**Table 6:** Antifungal activity of optimized CFF from PA 8–7 against 19 tropical crop pathogens.

Host Plant	Pathogen	Inhibition rate(%)
Rubber Tree	*C. gloeosporioides*	71.72 ± 1.02^d^
	*C. cassiicola*	47.76 ± 0.58^g^
	*C. alienum*	89.81 ± 0.44^b^
	*C. siamense*	85.88 ± 1.74^c^
	*A. heveae*	72.52 ± 1.65^d^
	*F. solani*	44.83 ± 1.72^h^
Nut Tree	*P. microspora*	83.77 ± 1.76^c^
	*C. pentaseptata*	36.49 ± 2.29^i^
Litchi	*P. litchii*	94.56 ± 1.38^a^
Melon	*P. melonis Katsura*	93.87 ± 1.69^a^
Cowpea	*P. vignae Purss*	86.29 ± 3.09^c^
Tomato	*A. solani*	35.80 ± 3.46^i^
Grape	*B. cinerea*	68.92 ± 3.16^e^
Tobacco	*A. nicotiana*	27.26 ± 4.68^g^
Pepper	*P. capsici*	22.42 ± 4.68^k^
Peach	*R. stolonifer*	22.05 ± 4.68^l^
Banana	*F.oxysporum* f. sp.*cubense*	6.78 ± 4.68^g^
Winter Jujube	*A. alternate*	51.69 ± 2.41^f^
Citrus	*D. passifloricola*	88.93 ± 0.54^b^

All the data are expressed as means ± SD of three independent experiments. Statistical analysis was performed using one-way ANOVA at *p* < 0.05 designated by superscripts a, b, c, d, e, f, g, h, i, j, k, and l.

### 3.5 Stability of the CFF

**Figure 7:**
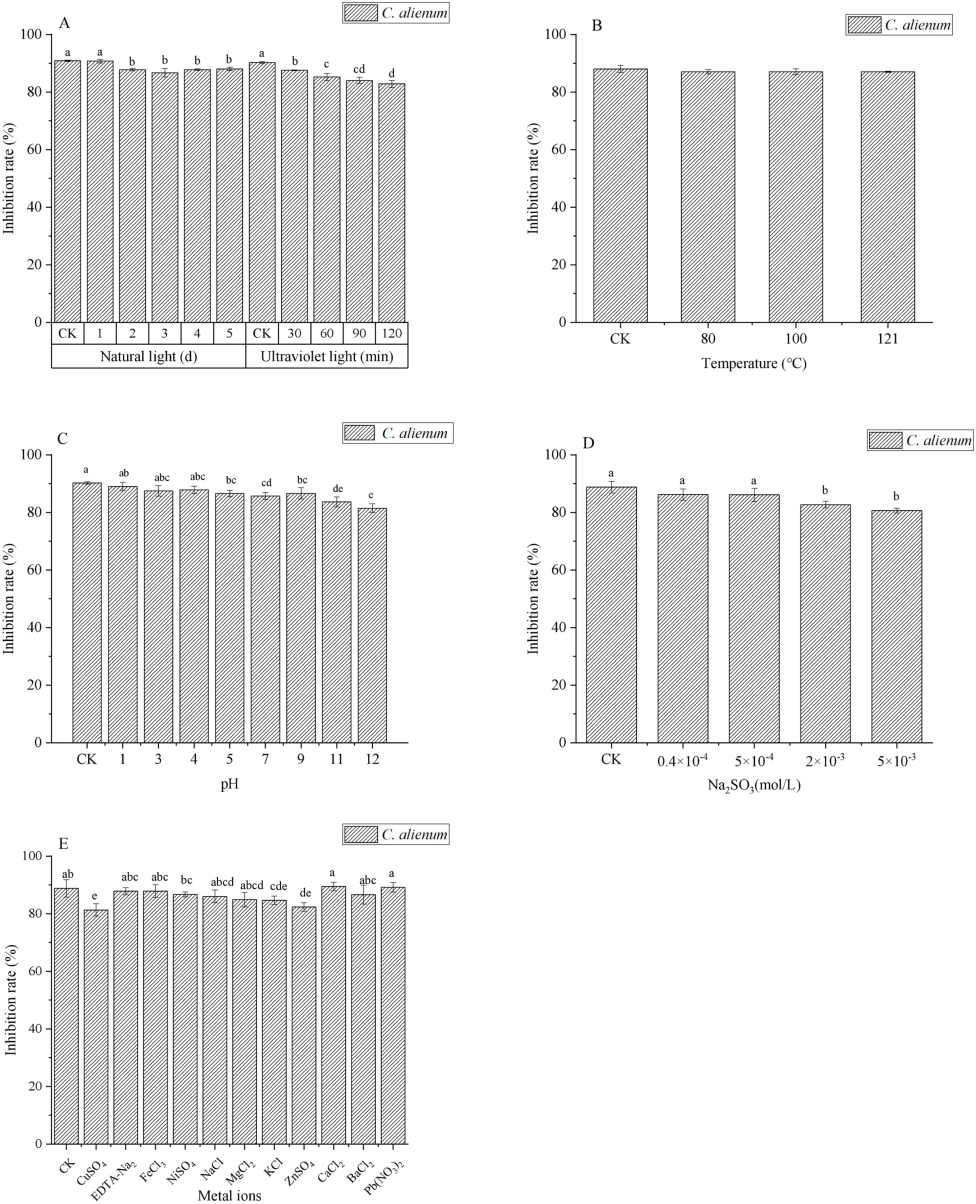
Environmental stability of the CFF. The antifungal activity of the CFF against *C. alienum* was measured after exposure to various stress conditions. Data are expressed as mean ± SD (n=3). **(A)** Light stability: UV irradiation for 30–120 min and natural light exposure for 1–5 days. The dashed line indicates the control (untreated CFF). **(B)** Thermal stability: incubation at 80 °C for 1 h, 100 °C for 1 h, and autoclaving at 121 °C for 30 min. **(C)** pH stability: CFF adjusted to pH 1–12 for 30 min, then neutralized before testing. **(D)** Reductant stability: CFF treated with increasing concentrations of Na_2_SO_3_ (0.4×10–^4^ to 5×10–^3^ mol/L). **(E)** Metal ion stability: CFF supplemented with various metal salts at a final concentration of 0.005 mol/L. Different letters above bars indicate statistically significant differences (one-way ANOVA, *p* < 0.05).

The CFF exhibited remarkable stability under various environmental stressors, as summarized in the multi-panel **Figure 7**.

**Figure 7A** illustrates the effect of light exposure. The CFF retained high antifungal activity after both UV irradiation and prolonged exposure to natural light. Even after 120 min of UV treatment, the inhibition rate remained at 82.82%, and after 5 days under natural light, activity was still 88.03% (compared to 90.88% for the control), indicating strong photostability.

Thermal stability was assessed by treating the CFF at different temperatures (**Figure 7B**). The antifungal activity remained virtually unchanged after incubation at 80 °C and 100 °C for 1 h, and even after autoclaving at 121 °C for 30 min, over 87% of the original activity was preserved (inhibition rate of 87.00% vs. 88.03% for the control), demonstrating exceptional heat resistance.

The CFF also exhibited excellent pH tolerance (**Figure 7C**). Under strongly acidic conditions (pH 1-4), the inhibition rate remained above 87%. Activity gradually decreased under alkaline conditions but still retained 81.49% at pH 12, confirming its stability over a wide pH range.

The effect of a reducing agent (Na_2_SO_3_) on CFF activity is shown in **Figure 7D**. At concentrations ≤5×10–^4^ mol/L, the inhibition rate was not significantly affected (≈86%). However, at higher concentrations (2×10–^3^ and 5×10–^3^ mol/L), the activity decreased slightly to 82.71% and 80.63%, respectively, indicating moderate tolerance to reducing conditions.

Finally, the impact of various metal ions on CFF stability was evaluated (**Figure 7E**). Most ions, including EDTA-Na_2_, FeCl_3_, NaCl, MgCl_2_, CaCl_2_, BaCl_2_, Pb(NO_3_)_2_, and NiSO_4_, had no significant adverse effect on antifungal activity. CuSO_4_ showed the strongest inhibition, yet the CFF still maintained 81.28% activity in the presence of 0.005 mol/L Cu^2+^. These results demonstrate that the CFF is highly compatible with a wide range of metal ions commonly encountered in agricultural.

### 3.6 The composition and content of rhamnolipids

**Figure 8:**
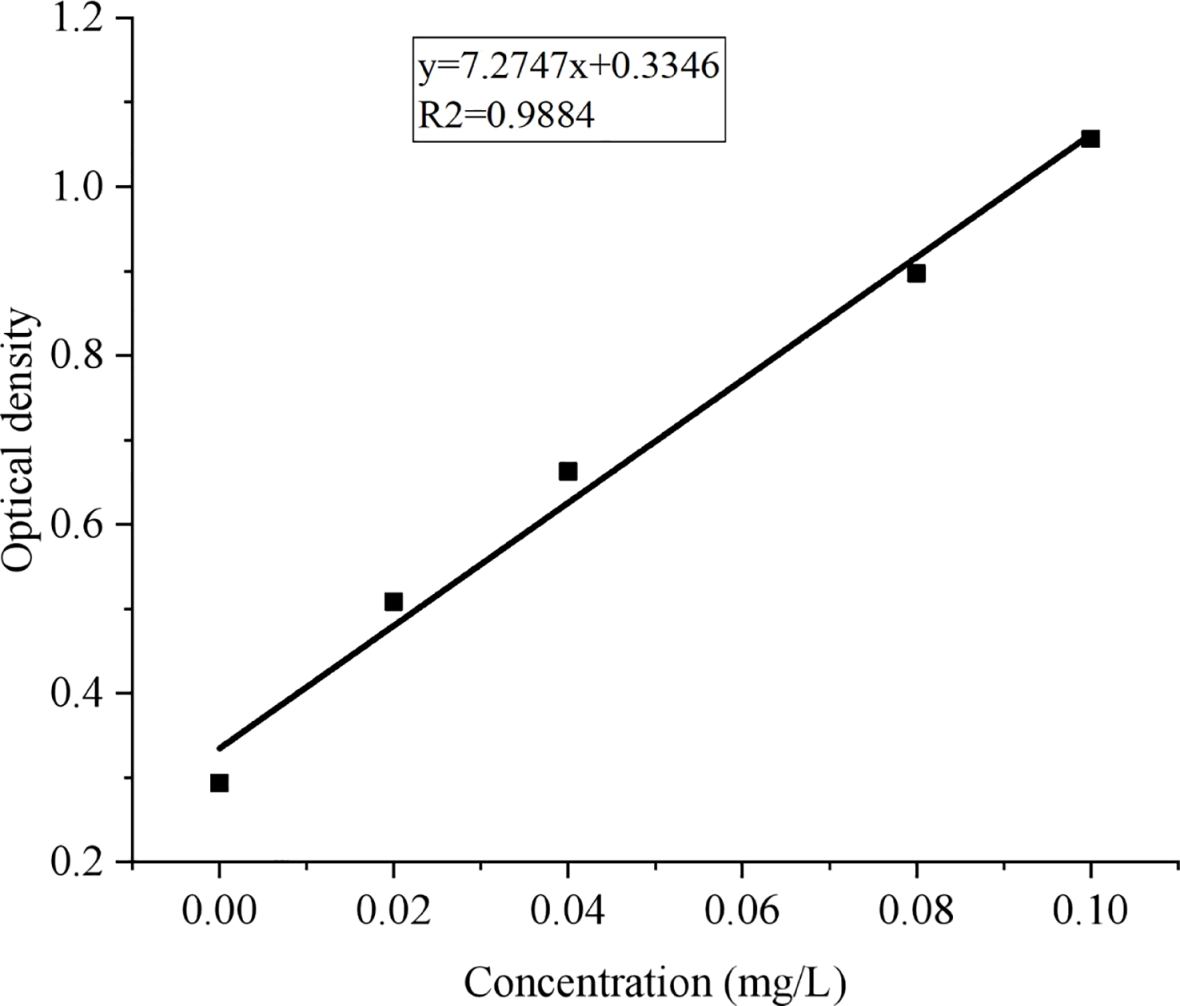
Standard curve for rhamnolipid quantification.

Rhamnolipids, the most extensively studied class of biosurfactants, are primarily produced by *P. aeruginosa* via fermentation. In this study, rhamnolipid concentration was quantified using the anthrone-sulfuric acid method, with a standard curve established as shown in **Figure 8**. Linear regression yielded the equation y=7.2747x+0.3346, with a coefficient of determination (R^2^ = 0.9884), indicating excellent linearity across the tested concentration range. While rhamnolipids were identified as the principal bioactive components in the CFF, it is important to note that the CFF is a complex mixture that may contain other secondary metabolites produced by *P. aeruginosa*, including phenazines, siderophores, and rhamnolipid biosynthetic precursors (HAAs). The rhamnolipid content expressed as rhamnose equivalents (determined by the anthrone-sulfuric acid method) was 13.97 g/L, representing approximately 26% of the fermentation broth dry weight. It is important to note that this method quantifies total sugars and may overestimate rhamnolipid concentrations due to interference from other carbohydrates (e.g., exopolysaccharides, lipopolysaccharides) present in the crude fermentation broth. Therefore, this value should be interpreted as a semi-quantitative estimate for comparative purposes, not as an absolute rhamnolipid yield. The relative contributions of other metabolites to the overall antifungal activity remain to be elucidated in future studies.

HPLC-MS analysis further revealed the compositional profile of the extracted rhamnolipids. By comparing retention times and characteristic [M-H]-ions (m/z) with those reported in the literature, a total of 18 rhamnolipid congeners were identified in the extract (**Table 7**, **Supplementary Figure 1**). These comprised 9 mono-rhamnolipids and 9 di-rhamnolipids. The structural features were further confirmed by ^1^HNMR spectroscopy (**Supplementary Figures 1**, **S2**). Due to the qualitative nature of the initial HPLC-MS analysis, the relative peak areas of individual congeners were not quantified. Nevertheless, the equal number of mono- and di-rhamnolipid congeners (9 each) identified in PA 8–7 suggests a potentially balanced distribution, which warrants further quantitative analysis.

**Table 7:** Rhamnolipid congeners identified in the fermentation broth of PA 8–7 by HPLC-MS analysis.

No	Num	m/z[M-H]-	Stru.	RT
1	Negpeak_group_000374	479.25	Rha-Rha-C10	7.51
2	Negpeak_group_000458	333.19	Rha-C10	7.96
3	Negpeak_group_000101	593.32	Rha-Rha-C8-C8	9.65
4	Negpeak_group_000236	447.26	Rha-C8-C8	10.30
5	Negpeak_group_000326 Di-RLs	475.29	Rha-C10-C8	11.69
6	Negpeak_group_000911	647.37	Rha-Rha-C8-C12:1	11.74
7	Negpeak_group_000714	475.29	Rha-C8-C10	11.76
8	Negpeak_group_000451 Di-RLs	675.40	Rha-Rha-C12:1-C10	12.66
9	Negpeak_group_000463 Di-RLs	649.38	Rha-Rha-C10-C10	12.69
10	Negpeak_group_001065	675.40	Rha-Rha-C10-C12:1	12.99
11	Negpeak_group_001032	503.32	Rha-C10-C10	13.26
12	Negpeak_group_000426 Di-RLs	677.40	Rha-Rha-C10-C12	13.44
13	Negpeak_group_000611	677.41	Rha-Rha-C12-C10	13.47
14	Negpeak_group_000129 Di-RLs	529.34	Rha-C12:1-C10	13.89
15	Negpeak_group_000534	529.34	Rha-C10-C12:1	13.95
16	Negpeak_group_001119	703.43	Rha-Rha-C12-C12:1	14.25
17	Negpeak_group_000518	531.35	Rha-C10-C12	14.31
18	Negpeak_group_000125	531.35	Rha-C12-C10	14.35

### 3.7 Safety evaluation of the CFF on soybean seedlings

**Figure 9:**
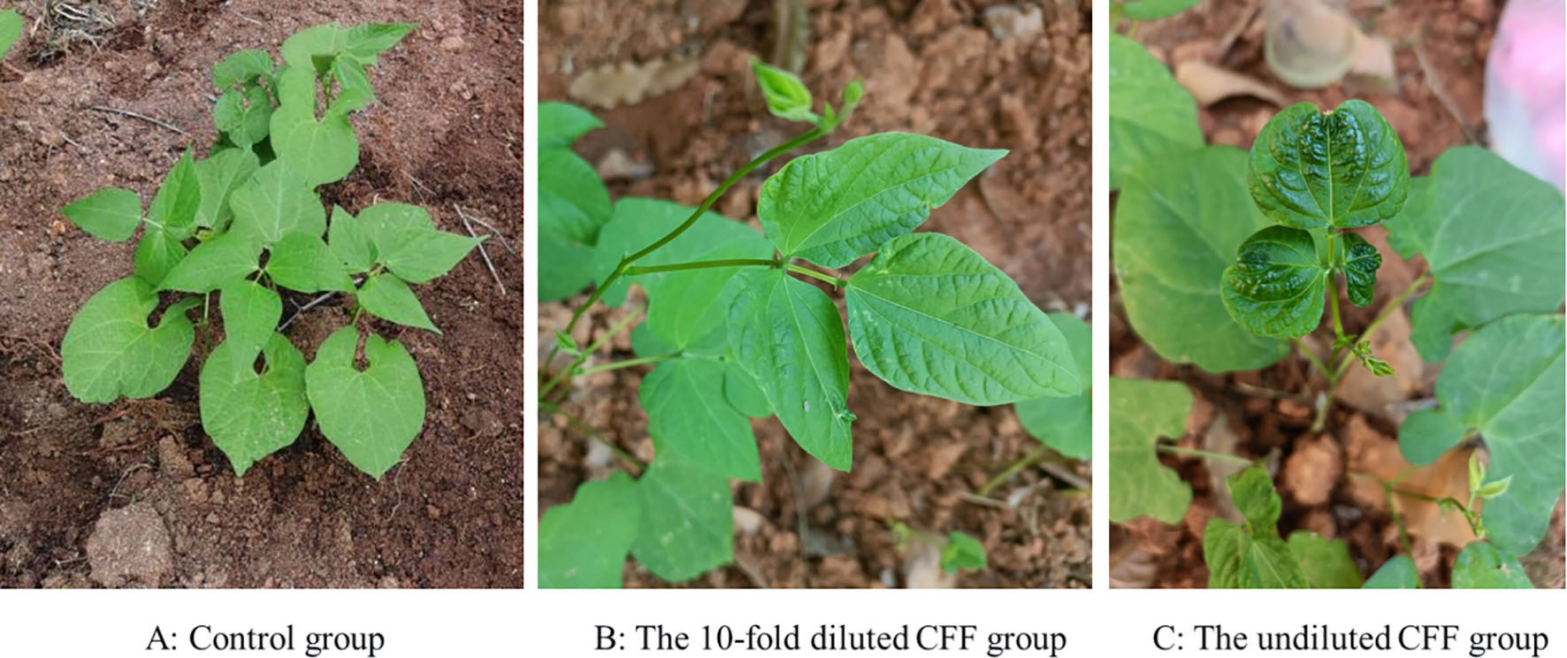
Safety evaluation on soybean seedlings.

After 7 days of foliar application, no obvious phytotoxicity was observed in any group (**Figure 9**). Seedlings in the control group (A) showed normal green, flat leaves with no chlorosis, necrosis, or deformation. The 10-fold diluted CFF group (B) exhibited growth status comparable to the control, with no abnormal symptoms. In the undiluted CFF group (C), seedlings maintained normal green color and overall healthy growth, but showed slight leaf curling and rounding, indicating a mild morphological effect at high concentration. No severe toxicity (e.g., wilting, necrosis, or stunting) was observed. These results suggest that the autoclaved CFF is safe for agricultural use at appropriate dilutions (e.g., 10-fold).

### 3.8 Field control efficacy against rubber tree powdery mildew

**Figure 10:**
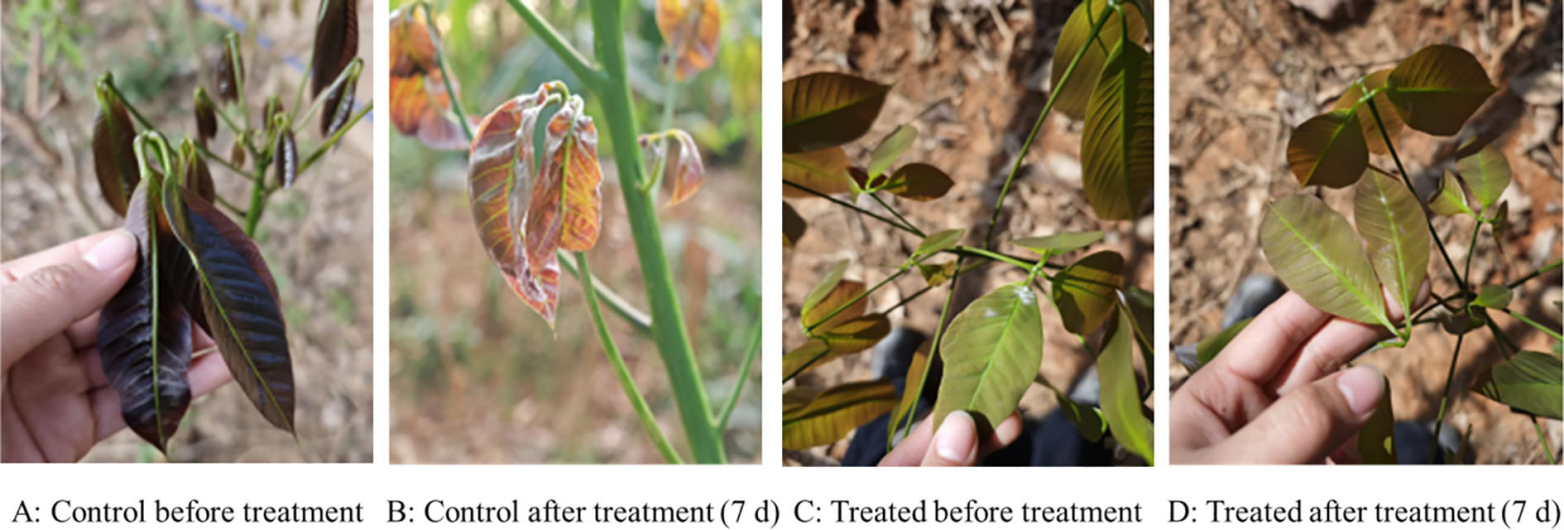
Field control efficacy against rubber tree powdery mildew.

As shown in **Figure 10** and **Table 8**, the disease index of the control group increased from 22.44 ± 3.45 to 75.33 ± 2.83 after 7 days, with leaves covered by white powdery mildew and severe curling (**Figures 10A, B**). In contrast, in the CFF−treated group, the disease index decreased from 23.55 ± 0.63 to 21.11 ± 0.31 within the same period, and powdery mildew lesions were visibly reduced (**Figures 10C, D**). The relative control efficacy of the 10−fold diluted CFF was 73.23% ± 4.80%. These data demonstrate that the CFF exhibits strong protective and curative effects against rubber tree powdery mildew under field conditions.

**Table 8:** Field control efficacy of the 10−fold diluted CFF against rubber tree powdery mildew.

Treatment	Disease index before treatment	Disease index after treatment	Control efficacy/%
CFF (10×diluted)	23.55 ± 0.63	21.11 ± 0.31	73.23 ± 4.80
Control (medium)	22.44 ± 3.45	75.33 ± 2.83	

## 4 Discussion

The production of antimicrobial metabolites by *Pseudomonas* is strongly influenced by carbon sources, yet this regulation is strain-dependent (Mendes et al., 2013; Zhang et al., 2025; De Vrieze et al., 2020). For instance, glucose promotes 2,4-diacetylphloroglucinol biosynthesis in some strains but not others (Wang et al., 2017; Zhang et al., 2018; Mishra et al., 2018; Chantal et al., 2015), and carbon sources also affect pyoluteorin and rhamnolipid composition (Cui et al., 2023; Zhu et al., 2017). This complexity justifies the independent optimization of PA 8-7. Although our orthogonal design identified optimal levels of casein hydrolysate, glycerol, K_2_HPO_4_ and MgSO_4_·7H_2_O, it does not capture interactions among components. Future use of response surface methodology could further enhance fermentation efficiency.

Despite the promise of *P. aeruginosa*−derived CFFs as biocontrol agents, several key gaps remain. Existing studies have used largely unoptimized fermentation conditions, characterized only minimal stability parameters, and relied almost exclusively on *in vitro* assays without field validation or biosafety assessment. The present study was designed to systematically address each of these gaps.

Our optimization increased the CFF’s inhibitory activity against *C. alienum* by 54% to 89.81%. The CFF exhibited remarkable stability, retaining >87% activity after autoclaving (121 °C), over pH 1–12, under UV irradiation, and in the presence of most metal ions, supporting its use in sterilization-requiring practices like seed coating.

Rhamnolipids, identified as major components (18 congeners by HPLC-MS), are EPA-approved biopesticides that disrupt fungal membranes (Kim et al., 2000; Müller et al., 2012). Their abundance and the strong correlation between rhamnolipid OD_625_ and antifungal activity suggest they are primary contributors. However, *P. aeruginosa* also produces phenazines, pyoluteorin, siderophores and volatile compounds (Pierson and Pierson, 2010; Cornelis and Dingemans, 2013). Without bioassay-guided fractionation or genetic knockout (e.g., ΔrhlAB), we cannot definitively exclude their contributions. Thus, our conclusion that rhamnolipids are the “principal bioactive constituents” should be viewed as a likelihood, not proven causality. The antimicrobial mechanisms of rhamnolipids include inhibiting spore germination and mycelial growth of *Phytophthora* and *Colletotrichum* species (Chen et al., 2021; Lahkar et al., 2017), and formulations like PRO1 have controlled *Phytophthora cryptogea* (Jonghe et al., 2005). Against bacteria, they disrupt membranes and cause lysis (Wu et al., 2023).

The retention of high antifungal activity (87%) after autoclaving (121 °C, 30 min) indicates that the biocontrol effect does not rely on live bacterial competition, quorum sensing, or heat−labile exoenzymes, but rather on heat−stable secondary metabolites. Genome analysis of PA 8−7 (GenBank PRJNA1199198) revealed gene clusters for phenazines, rhamnolipids, and siderophores. Phenazines can induce reactive oxygen species (ROS) causing oxidative damage; rhamnolipids are known to disrupt fungal membrane integrity (Kim et al., 2000; Chen et al., 2021); siderophores chelate iron, competing with pathogens for this nutrient. Based on these findings and previous reports, we propose that the CFF exerts antifungal activity through a synergistic combination of membrane disruption, oxidative stress, and iron competition. However, we did not experimentally investigate these mechanisms (e.g., ROS measurement, membrane permeability assays, or iron depletion tests), and the relative contribution of each pathway remains unknown. Direct experimental validation using gene knockout mutants or purified compounds will be the focus of future studies.

The CFF showed broad-spectrum activity against 19 tropical pathogens, with highest inhibition against *P. litchii* (94.56%) and *P. melonis* (93.87%), but weak activity against *F. oxysporum* f. sp. *cubense* (6.78%) and *P. capsici* (22.42%). This differential susceptibility may reflect interspecific variations in cell wall composition and membrane lipids (Chen et al., 2021). The resistance of *Fusarium* species aligns with reports that rhamnolipids are less effective against most *Ascomycetes* (Lahkar et al., 2017).

Our strain PA 8-7 produced equal numbers of mono- and di-rhamnolipid congeners (9 each). Their precise ratio remains unquantified, as our focus was on cost-effective fermentation rather than compositional optimization. In *P. aeruginosa*, RhlB produces mono-rhamnolipids from HAAs, and RhlC adds a second rhamnose to form di-rhamnolipids (Chong and Li, 2017). Different strains produce varying ratios; e.g., PAO1 produces 19 congeners (8 mono-, 12 di-) (Zhao et al., 2024), while strain 1.10452 produces nine with di-rhamnolipids predominant (Luo et al., 2022). Strategies to modulate mono/di ratios rely on strain selection, culture optimization and TLC purification (Samadi et al., 2012; Zhao et al., 2019), but homolog composition varies greatly among strains, and fatty acid chain characteristics affect performance (Robineau et al., 2020; Solaiman et al., 2015). The balanced distribution in PA 8-7 warrants further investigation for tailored formulations.

Comparison with existing CFF−based biocontrol studies. To articulate the contributions of this study relative to previous work, we compared our findings with representative reports (**Table 9**) (Di Rico et al., 2025; Kishore et al., 2005; Thammasittirong et al., 2025). Most previous studies used unoptimized media and reported only crude antifungal activity. In contrast, our orthogonal optimization increased the inhibition rate from 35.83% to 89.81% (54% enhancement). While rhamnolipids are generally stable up to 120 °C and pH 4–10, our CFF withstood autoclaving (121 °C) and extreme pH (1–12) with >87% activity retained. Tolerance to UV and 12 metal ions further distinguishes our CFF. Moreover, field validation of an autoclaved CFF against rubber tree powdery mildew (73.23% efficacy) with biosafety assessment has not been previously reported. These features—systematic optimization, exceptional stability, congener identification, and field efficacy with safety data—position our CFF as a practical, low−cost biocontrol candidate.

**Table 9:** Comparison of this study with representative *P. aeruginosa* CFF biocontrol studies.

Feature	SWUC02 (2025)	GSE 18 (2005)	SNTKU16 (2025)	This study
Fermentation optimization	×	×	×	√ (54% increase)
Extreme thermal stability (>100 °C)	×	×	×	✓
Wide pH tolerance (1–12)	×	×	×	✓
Metal ion/UV tolerance	×	×	×	✓
Rhamnolipid congener identification	×	×	×	√ (18 congeners)
Field efficacy validation	×	×	×	√ (73.23%efficacy)
Biosafety assessment (phytotoxicity)	×	×	×	√ (soybean, no toxicity)
Use of autoclaved, CFF	✓	× (live cells)	√ (but not autoclaved)	√ (autoclaved + field)

Field validation and safety. To address the lack of *in vivo* data, we evaluated the 10−fold diluted autoclaved CFF against rubber tree powdery mildew (*O. heveae*) under field conditions. Within 7 days, the disease index decreased from 23.55 to 21.11, with a relative control efficacy of 73.23%, comparable to or better than some commercial fungicides (e.g., triadimefon typically gives 60-70%). The same dilution caused no phytotoxicity on soybean seedlings, though undiluted CFF induced mild leaf curling, indicating a need for concentration optimization per crop. The CFF used in the field trial was autoclaved, confirming that its bioactive components remain functional after heat sterilization—a practical advantage for formulation and storage. To our knowledge, this is the first report of a crude, non-purified rhamnolipid-containing CFF from *P. aeruginosa* showing both biosafety and effective field control of a major tropical crop disease.

Biosafety considerations regarding the use of *P. aeruginosa*. A legitimate concern is the opportunistic pathogen status of *P. aeruginosa*. However, all experiments were performed with autoclaved, CFF containing no live bacteria. Autoclaving (121 °C, 30 min) kills all vegetative cells and denatures proteases, while rhamnolipids retain >87% activity. The CFF is intended for foliar application, not human consumption. Soybean seedlings showed no phytotoxicity at the working dilution (10-fold), and only mild leaf curling at undiluted concentration. For future large-scale use, producing rhamnolipids in non-pathogenic hosts (e.g., engineered *Pseudomonas chlororaphis*, *Bacillus subtilis*, or recombinant *E. coli*) could further enhance safety (Solaiman et al., 2015; Chong and Li, 2017).

Limitations. Several limitations should be acknowledged. (1) Kinetic parameters (biomass, substrate consumption) were not determined, hindering scale-up. (2) The anthrone-sulfuric acid method overestimates rhamnolipids due to cross-reactivity (Heyd et al., 2008; Behrens et al., 2016); thus, 13.97 g/L is rhamnose equivalents; accurate HPLC-MS/MS quantification is recommended. (3) Relative abundances of mono- and di-rhamnolipid congeners were not quantified. (4) Contributions from other antifungal compounds (e.g., phenazines) cannot be excluded; knockout studies (e.g., ΔrhlAB) are needed to establish causality. (5) Biomass and pH were not monitored in time-course experiments, preventing calculation of growth rates and yield coefficients. (6) We did not perform animal toxicity studies on the CFF or pathogenicity tests on the live strain. However, because the final product is sterile and cell-free, such tests are not essential for this applied study, though future registration as a biopesticide would require them. These limitations do not detract from the main findings but provide a roadmap for future work.

## 5 Conclusion

In conclusion, Unlike previous studies that used unoptimized culture conditions and lacked field validation, this study systematically optimized the fermentation process of PA 8–7 and, for the first time, evaluated the field efficacy of an autoclaved, CFF against rubber tree powdery mildew under real-world conditions. The optimal FA consisted of 15 g/L acid-hydrolyzed casein, 25 mL/L glycerol, 1 g/L MgSO_4_·7H_2_O and 1.5 g/L K_2_HPO_4_, with culture conditions set at 50 mL medium in a 500 mL flask, 220 rpm, 10% inoculum, 36 °C, and 5 days of incubation. The resulting CFF exhibited potent and broad-spectrum activity against key tropical crop pathogens, particularly *P. litchii* and *P. melonis*. Importantly, the CFF demonstrated exceptional environmental stability, retaining high activity after autoclaving, under extreme pH conditions (1–12), and upon exposure to UV light and most metal ions. Rhamnolipids were identified as major metabolites in the CFF; however, the filtrate also contains other secondary metabolites. Therefore, further bioassay-guided fractionation or genetic studies are required to conclusively determine the relative contribution of rhamnolipids to the overall antifungal efficacy. Notwithstanding this limitation, the remarkable stability and broad-spectrum activity of the CFF support its potential as a biocontrol agent for tropical agriculture. Field trials further confirmed that a 10-fold diluted CFF effectively controlled rubber tree powdery mildew (*Oidium heveae*) with 73.23% efficacy, without causing phytotoxicity, underscoring its practical promise as a sustainable biocontrol agent. The CFF itself combines high efficiency, broad-spectrum activity, and outstanding robustness, making it a strong candidate for the development of stable biocontrol formulations. Future work should focus on identifying the specific antimicrobial compounds responsible for the observed activity, scaling up the fermentation process, and further evaluating the efficacy and safety of the CFF under field conditions.

## Data Availability

The original contributions presented in the study are included in the article/**Supplementary Material**. Further inquiries can be directed to the corresponding author.
