# Supplementary material for: An optimized, rhamnolipid-containing cell-free filtrate from Pseudomonas aeruginosa 8–7 exhibits broad-spectrum antifungal activity and exceptional environmental stability
Source: Front Plant Sci. 2026 Jun 10;17:1809669. doi: 10.3389/fpls.2026.1809669 (PMC13290996; doi:10.3389/fpls.2026.1809669)
Supplement: Supplementary file 1 [file DataSheet1.zip › Supplementary files/Fig. S1.pdf]

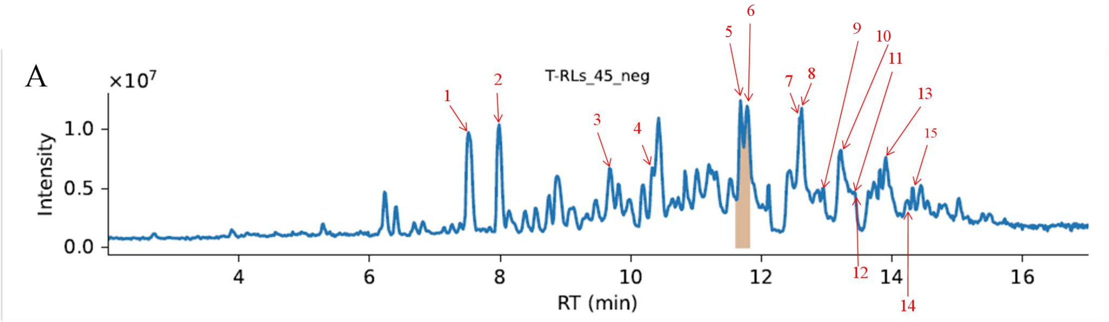


B

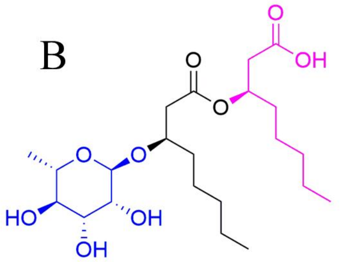

Rha-C8-C8

Chemical Formula: $C_{22}H_{40}O_9$
Exact Mass: 448.2672

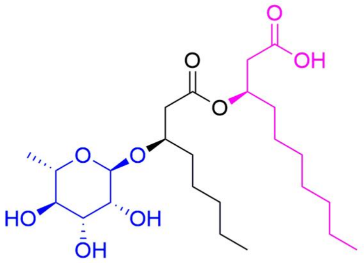

Rha-C8-C10

Chemical Formula: $C_{24}H_{44}O_9$
Exact Mass: 476.2985

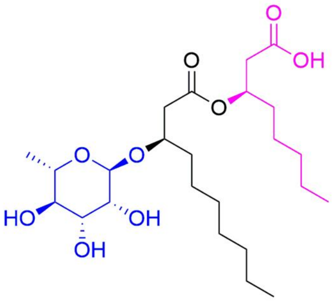

Rha-C10-C8

Chemical Formula: $C_{24}H_{44}O_9$
Exact Mass: 476.2985

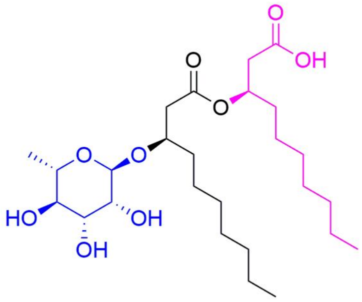

Rha-C10-C10

Chemical Formula: $C_{26}H_{48}O_9$
Exact Mass: 504.3298

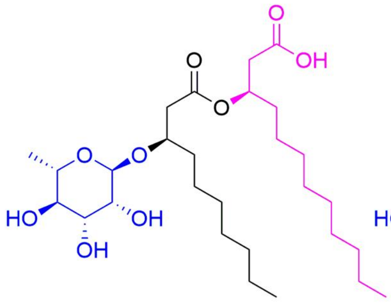

Rha-C10-C12

Chemical Formula: $C_{28}H_{52}O_9$
Exact Mass: 532.3611

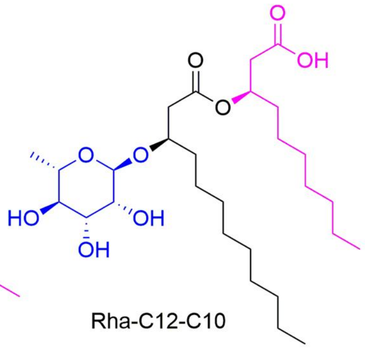

Rha-C12-C10

Chemical Formula: $C_{28}H_{52}O_9$
Exact Mass: 532.3611

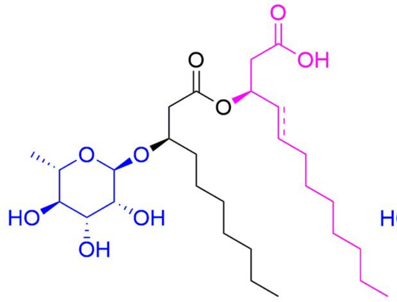

Rha-C10-C12:1

Chemical Formula: $C_{28}H_{50}O_9$
Exact Mass: 530.3455

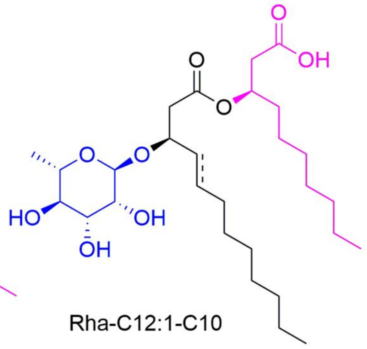

Rha-C12:1-C10

Chemical Formula: $C_{28}H_{50}O_9$
Exact Mass: 530.3455

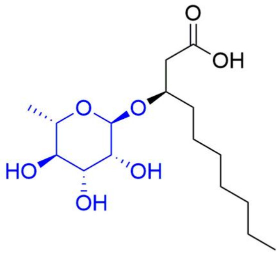

Rha-C10

Chemical Formula: $C_{16}H_{30}O_7$
Exact Mass: 334.1992

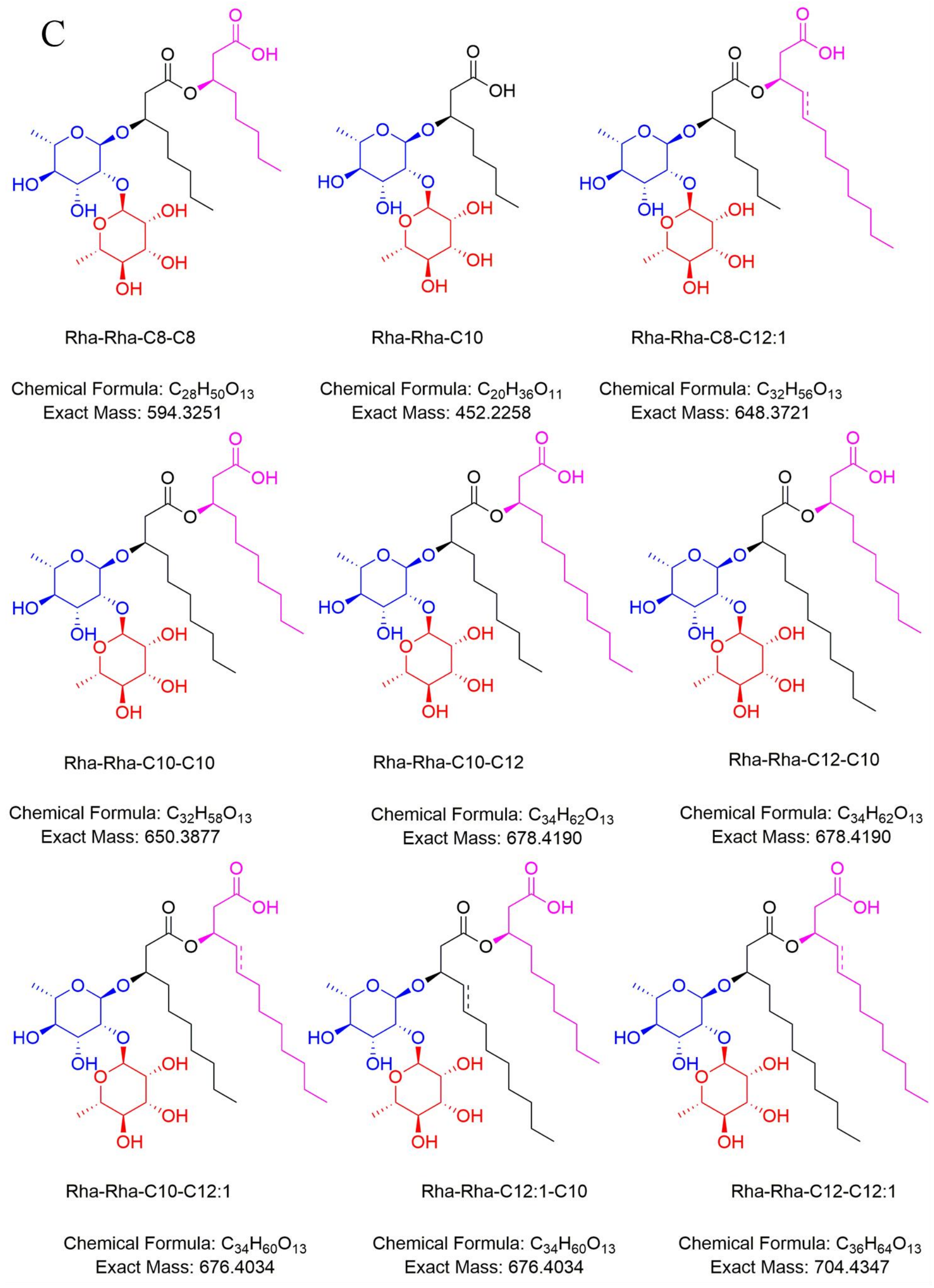


**Fig. S1** The composition of rhamnolipids

Representative images: 1: Rha-Rha-C10; 2: Rha-C10; 3: Rha-Rha-C8-C8; 4: Rha-C8-C8; 5: Rha-Rha-C8-C12:1; 6: Rha-C8-C10/Rha-C10-C8; 7: Rha-Rha-C12:1-C10; 8: Rha-Rha- C10-C10; 9: Rha-Rha-C10-C12:1; 10: Rha-C10-C10; 11: Rha-Rha-C10-C12; 12: Rha-Rha-C12-C10; 13: Rha-C10-C12:1/Rha-C12:1-C10; 14: Rha-Rha-C12-C12:1; 15: Rha-C10-C12/Rha-C12-C10.
