# Supplementary material for: An optimized, rhamnolipid-containing cell-free filtrate from Pseudomonas aeruginosa 8–7 exhibits broad-spectrum antifungal activity and exceptional environmental stability
Source: Front Plant Sci. 2026 Jun 10;17:1809669. doi: 10.3389/fpls.2026.1809669 (PMC13290996; doi:10.3389/fpls.2026.1809669)
Supplement: Supplementary file 1 [file DataSheet1.zip › Supplementary files/Fig. S2-di-rhamnolipids.pdf]

ecg757 H

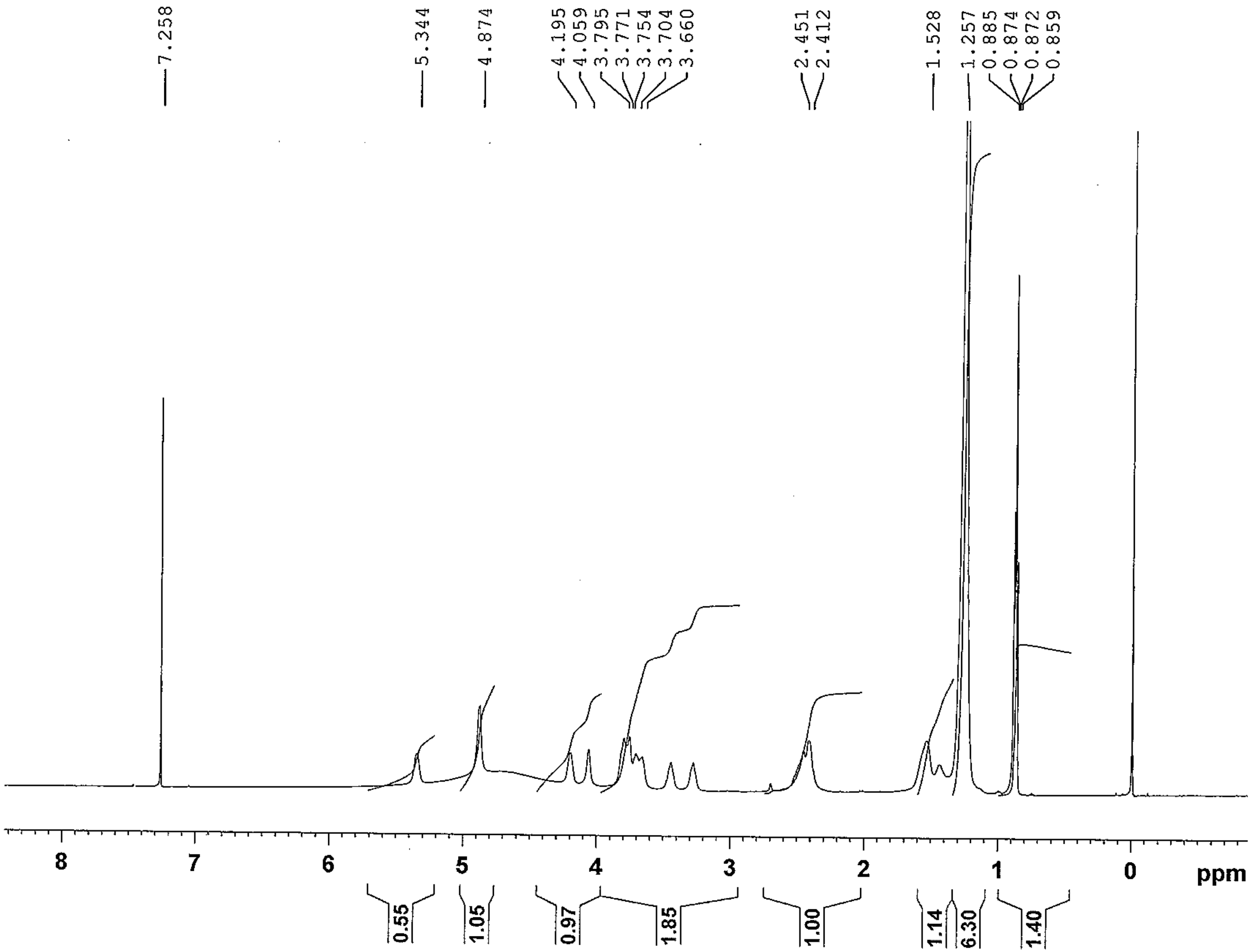

Current Data Parameters

NAME	ecg757
EXPNO	21
PROCNO	1

F2 - Acquisition Parameters

Date_	20240724
Time	10.21
INSTRUM	spect
PROBHD	5 mm CPQCI 1H/
PULPROG	zg
TD	65536
SOLVENT	CDCl3
NS	1
DS	0
SWH	10000.000 Hz
FIDRES	0.152588 Hz
AQ	3.2767999 sec
RG	30.61
DW	50.000 usec
DE	10.00 usec
TE	298.1 K
D1	1.00000000 sec
TD0	1

======== CHANNEL f1 ========

SFO1	500.1826280 MHz
NUC1	1H
P1	10.80 usec
PLW1	4.37519979 W

F2 - Processing parameters

SI	65536
SF	500.1800130 MHz
WDW	EM
SSB	0
LB	0.20 Hz
GB	0
PC	1.00

Kha-Kha-
