# Supplementary material for: An optimized, rhamnolipid-containing cell-free filtrate from Pseudomonas aeruginosa 8–7 exhibits broad-spectrum antifungal activity and exceptional environmental stability
Source: Front Plant Sci. 2026 Jun 10;17:1809669. doi: 10.3389/fpls.2026.1809669 (PMC13290996; doi:10.3389/fpls.2026.1809669)
Supplement: Supplementary file 1 [file DataSheet1.zip › Supplementary files/Fig. S2-mono-rhamnolipids.pdf]

ecg742 H

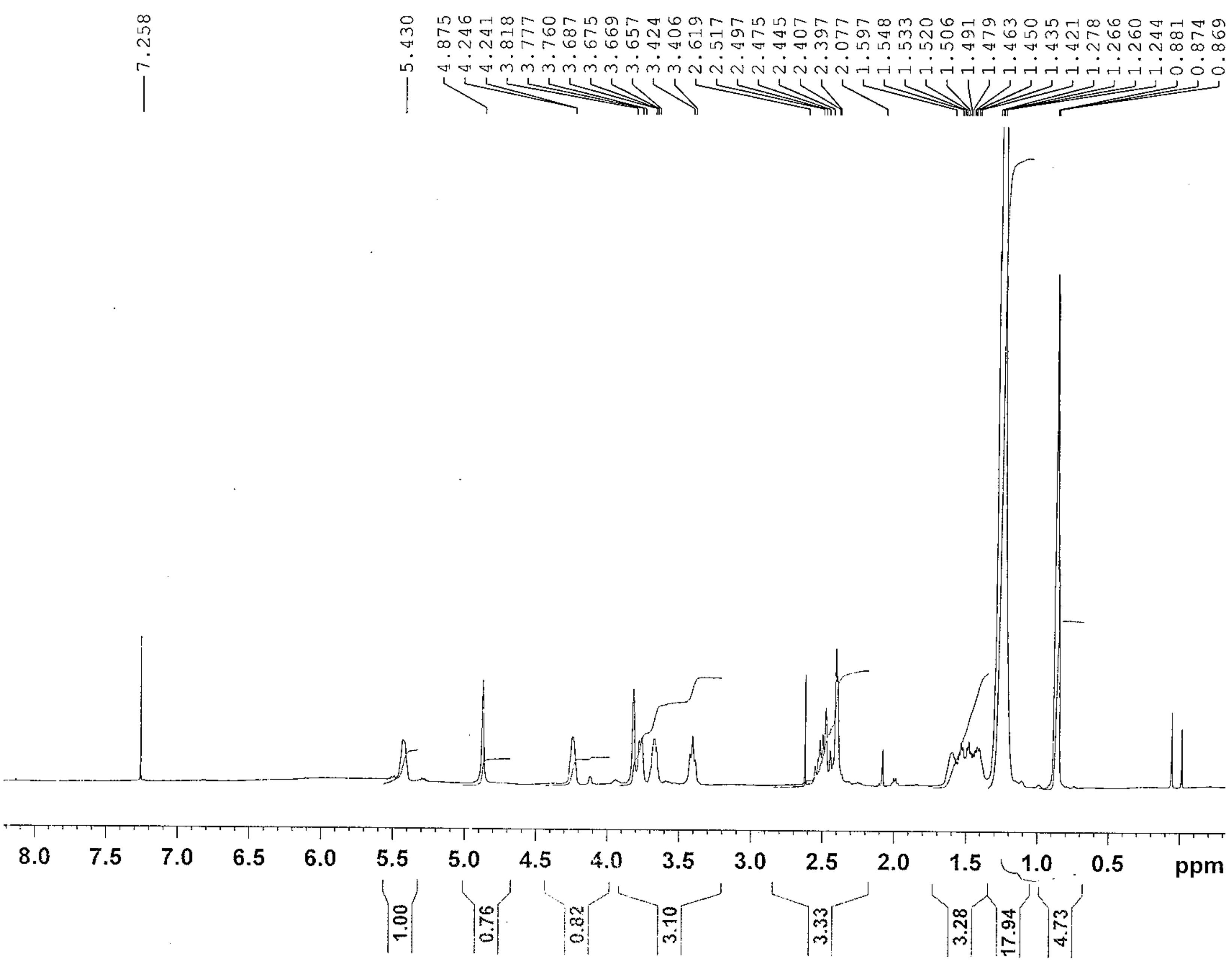

BRUKER

```
Current Data Parameters
NAME              ecg742
EXPNO                 21
PROCNO                 1

F2 - Acquisition Parameters
Date_           20240326
Time                9.19
INSTRUM            spect
PROBHD   5 mm CPQCI 1H/
PULPROG               zg
TD                 65536
SOLVENT            CDCl3
NS                     1
DS                     0
SWH             7500.000 Hz
FIDRES          0.114441 Hz
AQ             4.3690667 sec
RG                 30.61
DW                66.667 usec
DE                 10.00 usec
TE                 298.2 K
D1            2.00000000 sec
TD0                    1

======== CHANNEL f1 ========
SFO1        500.1825009 MHz
NUC1                  1H
P1                 10.80 usec
PLW1          4.37519979 W

F2 - Processing parameters
SI                 65536
SF          500.1800130 MHz
WDW                   EM
SSB      0
LB                  0.10 Hz
GB       0
PC                  1.00
```
