# Supplementary material for: An optimized, rhamnolipid-containing cell-free filtrate from Pseudomonas aeruginosa 8–7 exhibits broad-spectrum antifungal activity and exceptional environmental stability
Source: Front Plant Sci. 2026 Jun 10;17:1809669. doi: 10.3389/fpls.2026.1809669 (PMC13290996; doi:10.3389/fpls.2026.1809669)
Supplement: Supplementary file 1 [file DataSheet1.zip › Supplementary files/Supplementary Methods.docx]

2.9 Extraction and Determination of Rhamnolipids

2.9.1 Rhamnolipid Quantification by Anthrone‑Sulfuric Acid Method

Rhamnolipid concentration in the fermentation broth was determined using the anthrone‑sulfuric acid method (Qi and Xu, 2010). The principle of this method is based on acid hydrolysis of rhamnolipids upon heating with concentrated sulfuric acid, which releases L‑rhamnose. The released rhamnose then reacts with anthrone to form a blue‑green complex, the absorbance of which is proportional to the rhamnose concentration. The rhamnolipid concentration is subsequently calculated using a predetermined conversion factor.

Construction of the L-Rhamnose Standard Curve: Anthrone-sulfuric acid reagent (0.2% w/v) was freshly prepared by dissolving 0.2 g of anthrone in 100 mL of concentrated sulfuric acid. An L‑rhamnose stock solution (1 g/L) was prepared by dissolving 0.01 g of L‑rhamnose in 10 mL of distilled water. This stock solution was serially diluted with distilled water to produce a series of standard solutions at concentrations of 0, 20, 40, 60, 80, and 100 mg/L. For each standard, 1.25 mL was transferred to a 5 mL centrifuge tube. Under ice‑bath conditions, 2.5 mL of the freshly prepared anthrone‑sulfuric acid reagent was slowly added along the tube wall to prevent excessive heat generation. After thorough mixing, the tube was sealed and incubated in a boiling water bath for exactly 15 min. Following incubation, the mixture was cooled to room temperature. An aliquot of 200 μL from each reaction mixture was transferred to a 96‑well plate, and the absorbance was measured at 625 nm using a microplate reader (BioTek Instruments, Inc., USA). The standard curve was plotted with L‑rhamnose concentration (mg/L) on the x‑axis and absorbance at 625 nm on the y‑axis. Linear regression analysis was performed to obtain the calibration equation.

Determination of rhamnolipid concentration in fermentation broth: To optimize fermentation conditions, the effects of inoculum size (5%, 10%, 15%, 20%, 25%, v/v), culture temperature (30, 33, 36, 39 °C), shaker speed (120, 140, 160, 180, 220 rpm), culture time (4, 5, 6, 7, 8 d), and liquid volume in 500 mL flasks (50, 100, 150, 200 mL) on rhamnolipid synthesis and antifungal activity were systematically investigated. After fermentation, two parameters were simultaneously determined: the absorbance at 625 nm (OD₆₂₅) measured by the anthrone‑sulfuric acid method to reflect the relative accumulation level of rhamnolipids, and the inhibition rate of the supernatant to comprehensively evaluate the synthesis efficiency and functional activity of the fermentation products under different conditions. Based on these results, appropriate levels of each factor were determined.

In both the single‑factor experiments and subsequent validation under optimal conditions, rhamnolipid content was determined using the same anthrone‑sulfuric acid method. The detailed procedure was as follows: 2 mL of fermentation broth was centrifuged at 12,000 rpm for 10 min at room temperature. The supernatant was then diluted 15‑fold by mixing 200 μL of supernatant with 1.8 mL of distilled water, followed by removal of 500 μL of the mixed solution. Then, 1.25 mL of the diluted solution was transferred into a tube, and 2.5 mL of 0.2% anthrone‑sulfuric acid reagent was added slowly in an ice‑water bath. After mixing, the tube was heated in a boiling water bath for 15 min and then cooled to room temperature. Subsequently, 200 μL of the reaction solution was transferred into a 96‑well plate, and the absorbance was measured at 625 nm. The equivalent rhamnolipid concentration was calculated using an L‑rhamnose standard curve, and the actual rhamnolipid content was derived based on a conversion factor and the total dilution factor.

Based on the single‑factor experimental results, the optimal fermentation conditions were determined. Validation cultures were then performed using FA medium (containing 15 g/L acid‑hydrolyzed casein (Cat. No. C822594), 25 mL/L glycerol, 1 g/L MgSO₄·7H₂O, and 1.5 g/L K₂HPO₄, natural pH) under the following conditions: 50 mL of medium in a 500 mL flask, 10% (v/v) inoculum size, 36 °C, 220 rpm, and 5 days of incubation. Rhamnolipid content in the fermentation broth under these conditions was determined using the same procedure described above.

2.9.2 Extraction of Rhamnolipids

Rhamnolipids were extracted from the cell‑free fermentation filtrate using the acid precipitation method (Shuang et al., 2017), with modifications. Briefly, 50 mL of fermentation supernatant was collected, and its pH was adjusted to 2.0 by dropwise addition of 6 mol/L HCl with constant stirring. The acidified supernatant was incubated overnight at 4 °C to allow complete precipitation of rhamnolipids. The precipitate was collected by centrifugation at 12,000 r/min (approximately 13,400×g) for 10 min at 4 °C. The supernatant was discarded, and the precipitate was subjected to extraction three times with a mixed solvent of ethyl acetate and methanol (2:1, v/v). For each extraction, the precipitate was resuspended in 20 mL of the solvent mixture, vigorously shaken for 30 min, and centrifuged at 12,000 r/min for 10 min at 4 °C. The organic phases from the three extractions were combined and adjusted to pH 7.0 using 1 mol/L NaOH to neutralize any residual acid. The combined extract was centrifuged again under the same conditions to remove any insoluble impurities. The supernatant was filtered through a 0.22 μm organic‑compatible membrane filter (MilliporeSigma, USA) to ensure removal of particulate matter. The filtrate was transferred to a round‑bottom flask, and the solvent was removed under reduced pressure at 40 °C using a rotary evaporator (Büchi Labortechnik AG, Switzerland). The obtained crude rhamnolipid product was weighed to determine the extraction yield.

2.9.3 Rhamnolipid Component Analysis

The crude rhamnolipid extract was subjected to qualitative analysis using thin‑layer chromatography (TLC) and high‑performance liquid chromatography–mass spectrometry (HPLC–MS) to identify the individual rhamnolipid congeners.

Thin‑Layer Chromatography (TLC). TLC was performed on silica gel 60 F₂₅₄ aluminum plates (20 cm × 20 cm, 0.2 mm thickness; Merck KGaA, Germany). The crude rhamnolipid extract was dissolved in methanol at a concentration of 10 mg/mL, and 5–10 μL of the solution was spotted onto the TLC plate using a glass capillary tube. The plate was developed in a chromatographic chamber pre‑saturated with a mobile phase consisting of chloroform:methanol:acetic acid (65:15:2, v/v/v). After development, the plate was air‑dried, and spots were visualized by spraying with 50% sulfuric acid followed by heating at 120 °C for 10 min. Rhamnolipids appeared as brownish‑yellow spots against a light background.

High‑Performance Liquid Chromatography–Mass Spectrometry (HPLC–MS). For detailed compositional analysis, HPLC–MS was performed using an Agilent 1290 Infinity II UPLC system coupled with an Agilent 6545 Q‑TOF mass spectrometer equipped with an electrospray ionization (ESI) source (Agilent Technologies, USA).

Chromatographic Conditions. Separation was carried out on an ACQUITY UPLC HSS T3 column (2.1 mm × 100 mm, 1.8 μm particle size; Waters Corporation, USA) maintained at 40 °C. The autosampler temperature was set at 10 °C, and the injection volume was 2 μL. Mobile phase A consisted of water containing 0.04% (v/v) acetic acid, and mobile phase B was acetonitrile (HPLC grade). The flow rate was maintained at 0.35 mL/min. The gradient elution program was as follows: 0–0.5 min, 2% B; 0.5–15 min, linear increase from 2% to 98% B; 15–17 min, hold at 98% B; 17.1–20 min, return to 2% B and re‑equilibrate.

Mass Spectrometry Conditions. Mass spectrometry detection was performed in negative ion mode, as rhamnolipids form stable [M–H]⁻ ions under these conditions. The ion source parameters were optimized as follows: drying gas (nitrogen) temperature, 300 °C; drying gas flow rate, 8 L/min; nebulizer pressure, 35 psi; sheath gas temperature, 350 °C; sheath gas flow rate, 11 L/min; capillary voltage, 4000 V; nozzle voltage, 500 V; fragmentor voltage, 150 V; skimmer voltage, 65 V. Data were acquired in data‑dependent acquisition (DDA) mode over a mass range of 100–1200 m/z at a scan rate of 6 Hz. For each full MS scan, five subsequent MS/MS scans were triggered for the most abundant precursor ions. The isolation width for precursor ions was approximately 1.3 m/z, and a collision energy of 45 eV was applied for fragmentation.

Data Analysis. Rhamnolipid congeners were identified by comparing their retention times and characteristic [M–H]⁻ ions with those reported in the literature (Bai et al., 2012; Zhao et al., 2024; Luo et al., 2022). For structural verification of selected congeners, ¹H‑NMR spectra were recorded on a Bruker DRX‑500 spectrometer (Bruker, Karlsruhe, Germany) operating at 500 MHz. Samples were dissolved in deuterated chloroform (CDCl₃) or deuterated methanol (CD₃OD), and chemical shifts were referenced to tetramethylsilane (TMS) as an internal standard.

Column Chromatography (for NMR sample preparation). Silica gel (200–300 mesh, Qingdao Marine Chemical Inc., China), Chromatorex C18 (40–75 μm, Fuji Silysia Chemical Ltd., Japan), and Sephadex LH-20 (GE Healthcare Bio-Sciences AB, Uppsala, Sweden) were used as stationary phases. Fractions were monitored by TLC, and those containing the target compounds were pooled and concentrated.
