# Supplementary material for: An optimized, rhamnolipid-containing cell-free filtrate from Pseudomonas aeruginosa 8–7 exhibits broad-spectrum antifungal activity and exceptional environmental stability
Source: Front Plant Sci. 2026 Jun 10;17:1809669. doi: 10.3389/fpls.2026.1809669 (PMC13290996; doi:10.3389/fpls.2026.1809669)
Supplement: Supplementary file 1 [file DataSheet1.zip › Supplementary files/Table S1.pdf]

Table S1. List of chemical reagents used in this study.

Reagent	Catalog Number	Manufacturer
Anhydrous ethanol	A631029	Sangon Biotech Co., Ltd
Yeast extract	A515245	
Tryptone	A110859	
NaCl	B548119	
K_2HPO_4	A610438	
KH_2PO_4	A610498	
$MgSO_4 \cdot 7H_2O$	A610449	
Potassium nitrate	A610460	
Sucrose	A610498	
Peptone	A505247	
Beef extract	A505246	
Soluble starch	A610502	
Glycerol	A100854	
$CuSO_4$	A466012	
$FeCl_3$	A600454	
$NiSO_4$	A603034	
$MgCl_2$	A601336	
KCl	A100395	
$ZnSO_4$	A602906	
$CaCl_2$	A100556	
$BaCl_2$	A501162	
$Pb(NO_3)_2$	A502732	
Casein acid hydrolysate	C8221	Solarbio Science & Technology Co., Ltd., Beijing.
Glucose	G1160	
Maltose	M1000	
Lactose	L1000	
Galactose	G1000	
Fructose	F1000	
EDTA-Na_2	E8030	
Casein acid hydrolysate	C822594	Macklin Biochemical Co., Ltd., Shanghai.
Mineral oil	S30005	Yuanye Bio-Technology Co., Ltd
Soybean oil	S24362	
Corn oil	S50856	
Peanut oil	S27176	
Rapeseed oil	S54002	
Olive oil	S30503	
Palm oil	S25824	
Xylose	T92938	
Mannitol	V5667	
Soybean meal	Y030A	Beijing Hongrun Baoshun Technology Co., Ltd.
Corn steep liquor	Y042T	
