# Supplementary material for: An optimized, rhamnolipid-containing cell-free filtrate from Pseudomonas aeruginosa 8–7 exhibits broad-spectrum antifungal activity and exceptional environmental stability
Source: Front Plant Sci. 2026 Jun 10;17:1809669. doi: 10.3389/fpls.2026.1809669 (PMC13290996; doi:10.3389/fpls.2026.1809669)
Supplement: Supplementary file 1 [file DataSheet1.zip › Supplementary files/Table S2.pdf]

Table S2. Rhamnolipid accumulation (OD_{625}) under different single-factor fermentation conditions.

Parameter	Level	OD_{625}
Inoculum size (%)	5	1.22 ± 0.13
	10	1.61 ± 0.01
	15	1.445 ± 0.01
	20	0.93 ± 0.01
	25	0.86 ± 0.02
Culture Temperature (°C)	30	0.59 ± 0.00
	33	1.27 ± 0.01
	36	1.68 ± 0.01
	39	0.93 ± 0.03
Rotational speed (rpm)	120	0.59 ± 0.01
	140	0.67 ± 0.01
	160	1.37 ± 0.04
	180	1.55 ± 0.01
	220	1.93 ± 0.01
Culture time (day)	4	1.98 ± 0.03
	5	2.27 ± 0.02
	6	2.32 ± 0.01
	7	2.42 ± 0.04
	8	2.12 ± 0.04
Liquid volume (mL per 500 mL flask)	50	2.34 ± 0.02
	100	1.94 ± 0.01
	150	1.55 ± 0.04
	200	1.26 ± 0.02
