# Supplementary figures and images for: An optimized, rhamnolipid-containing cell-free filtrate from Pseudomonas aeruginosa 8–7 exhibits broad-spectrum antifungal activity and exceptional environmental stability
Source: Front Plant Sci. 2026 Jun 10;17:1809669. doi: 10.3389/fpls.2026.1809669 (PMC13290996; doi:10.3389/fpls.2026.1809669)

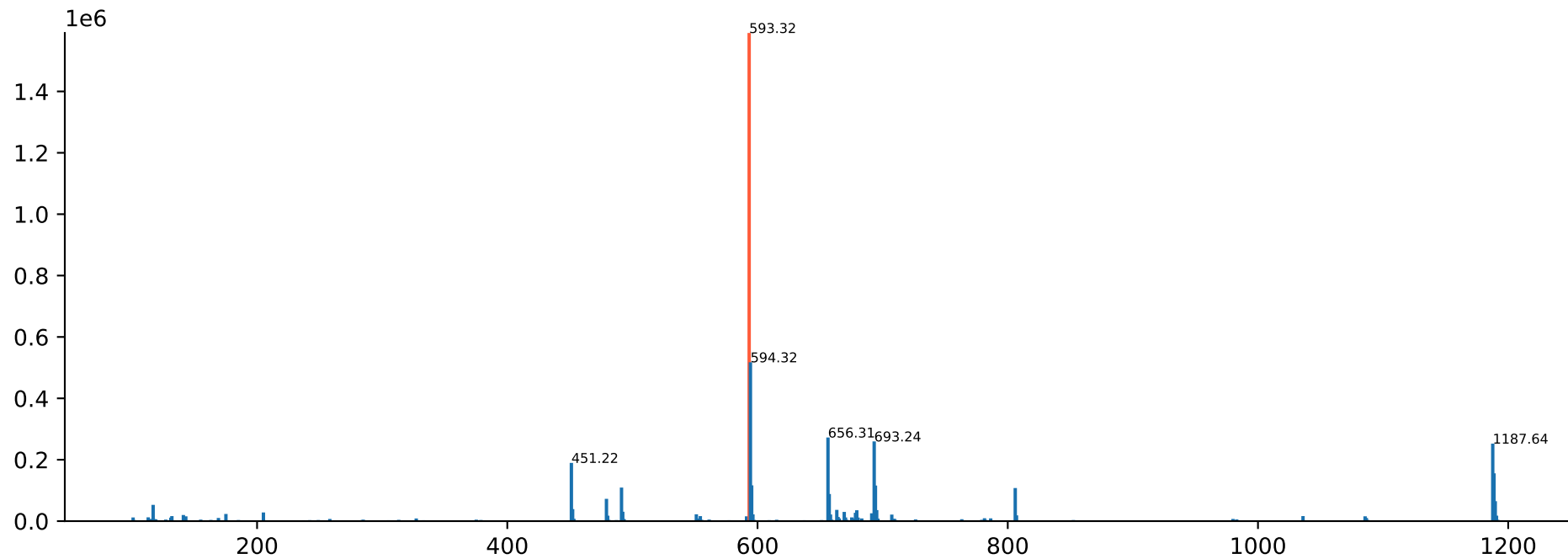

Supplement: Supplementary file 1 [file DataSheet1.zip › Supplementary files/Rhamnolipid Components in Fermentation Broth/Negpeak_group_000101.pdf]

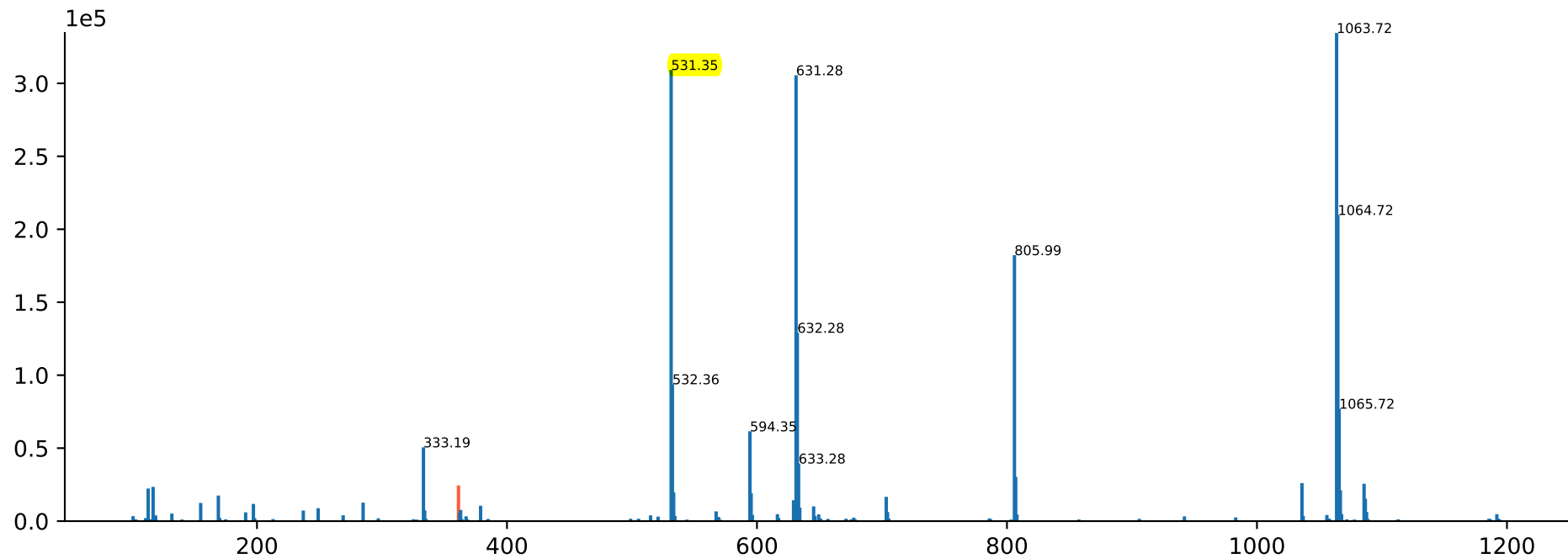

Supplement: Supplementary file 1 [file DataSheet1.zip › Supplementary files/Rhamnolipid Components in Fermentation Broth/Negpeak_group_000125.pdf]

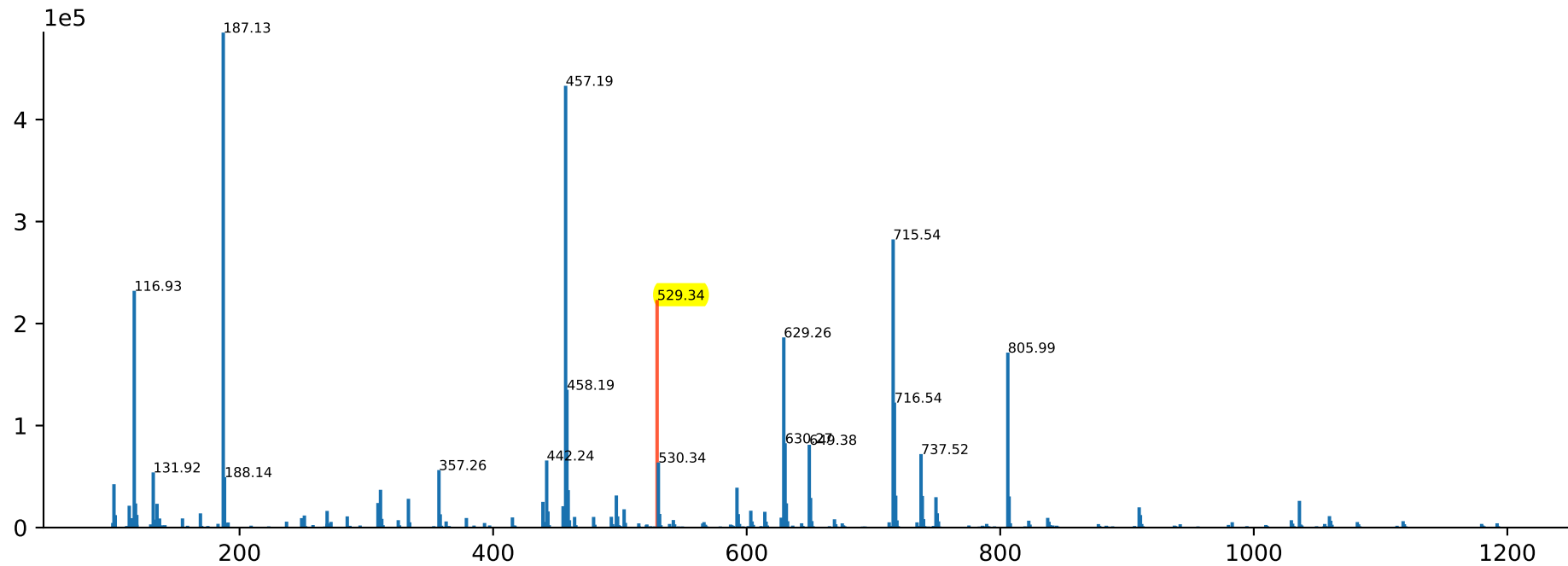

Supplement: Supplementary file 1 [file DataSheet1.zip › Supplementary files/Rhamnolipid Components in Fermentation Broth/Negpeak_group_000129.pdf]

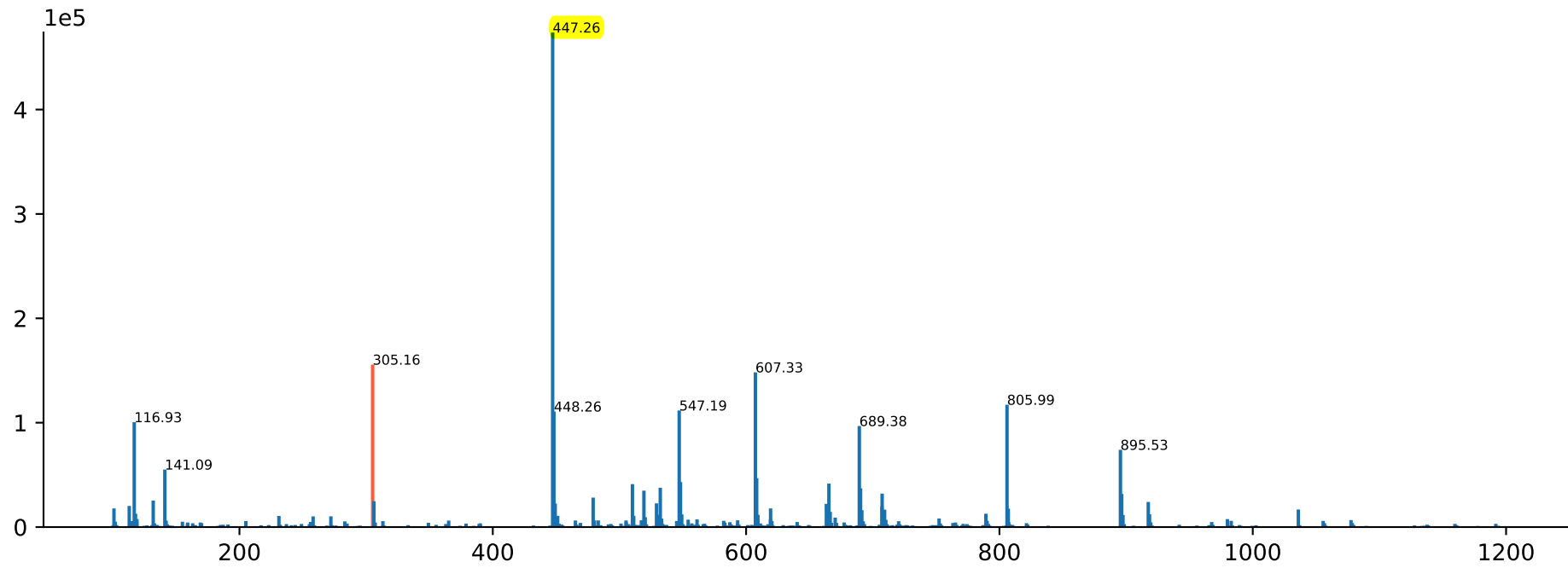

Supplement: Supplementary file 1 [file DataSheet1.zip › Supplementary files/Rhamnolipid Components in Fermentation Broth/Negpeak_group_000236.pdf]

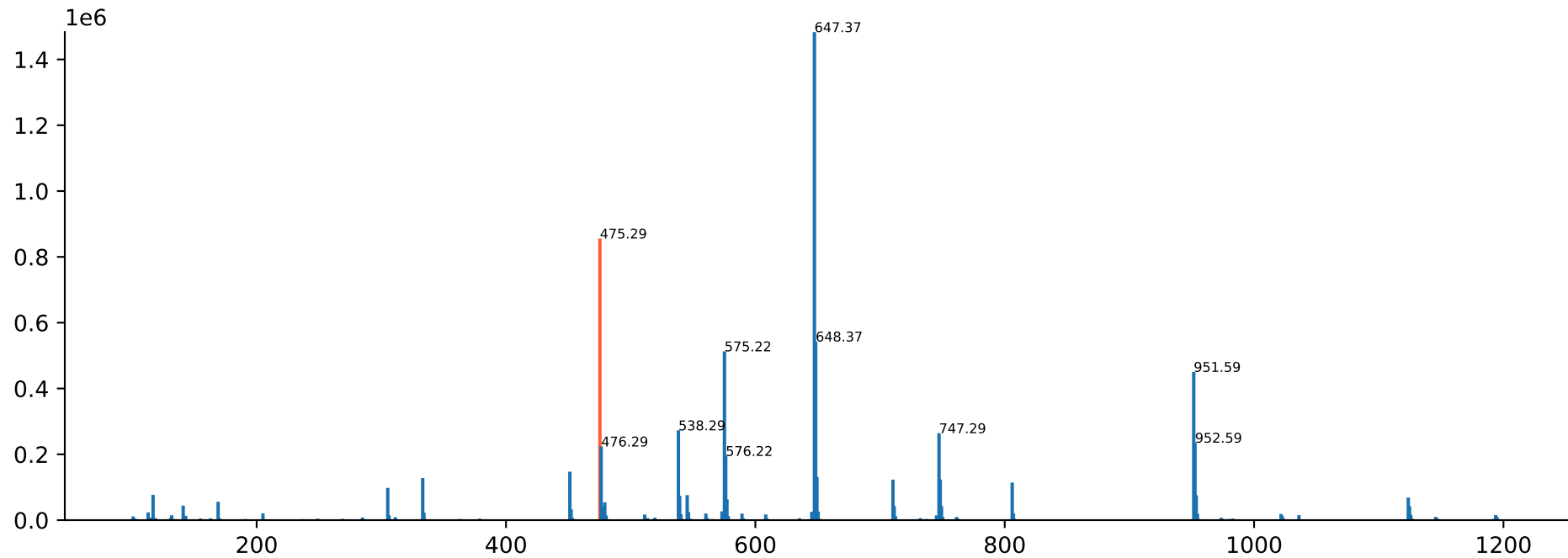

Supplement: Supplementary file 1 [file DataSheet1.zip › Supplementary files/Rhamnolipid Components in Fermentation Broth/Negpeak_group_000326.pdf]

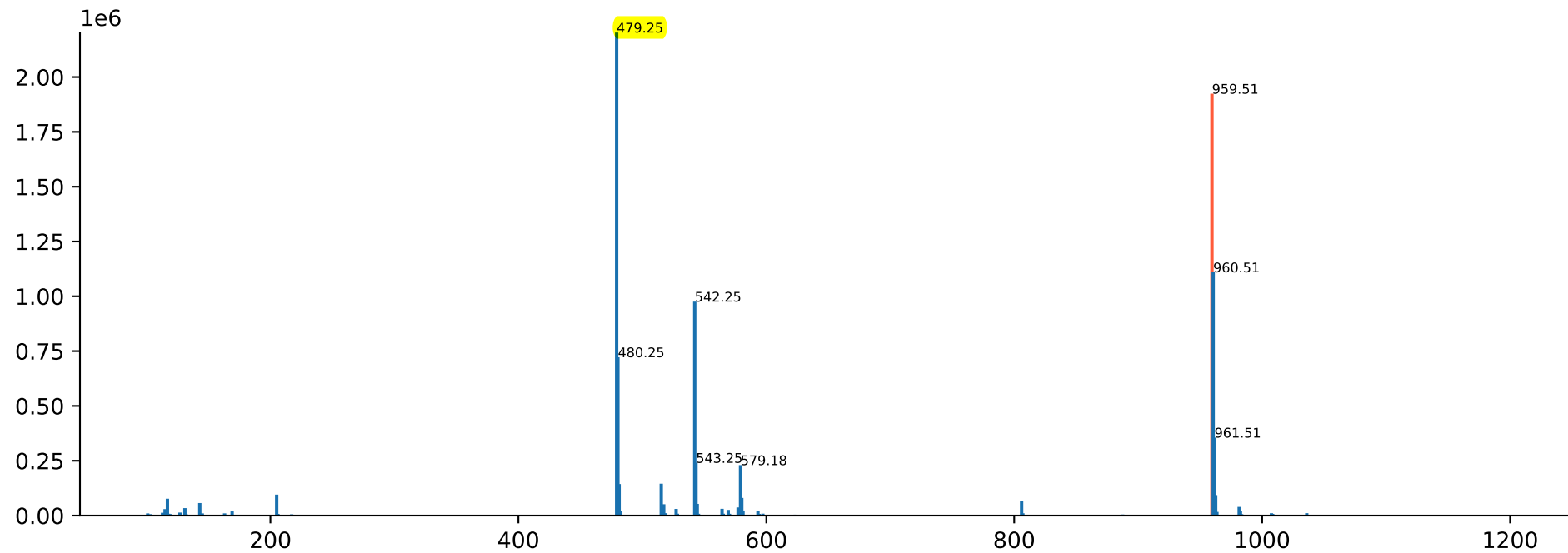

Supplement: Supplementary file 1 [file DataSheet1.zip › Supplementary files/Rhamnolipid Components in Fermentation Broth/Negpeak_group_000374.pdf]

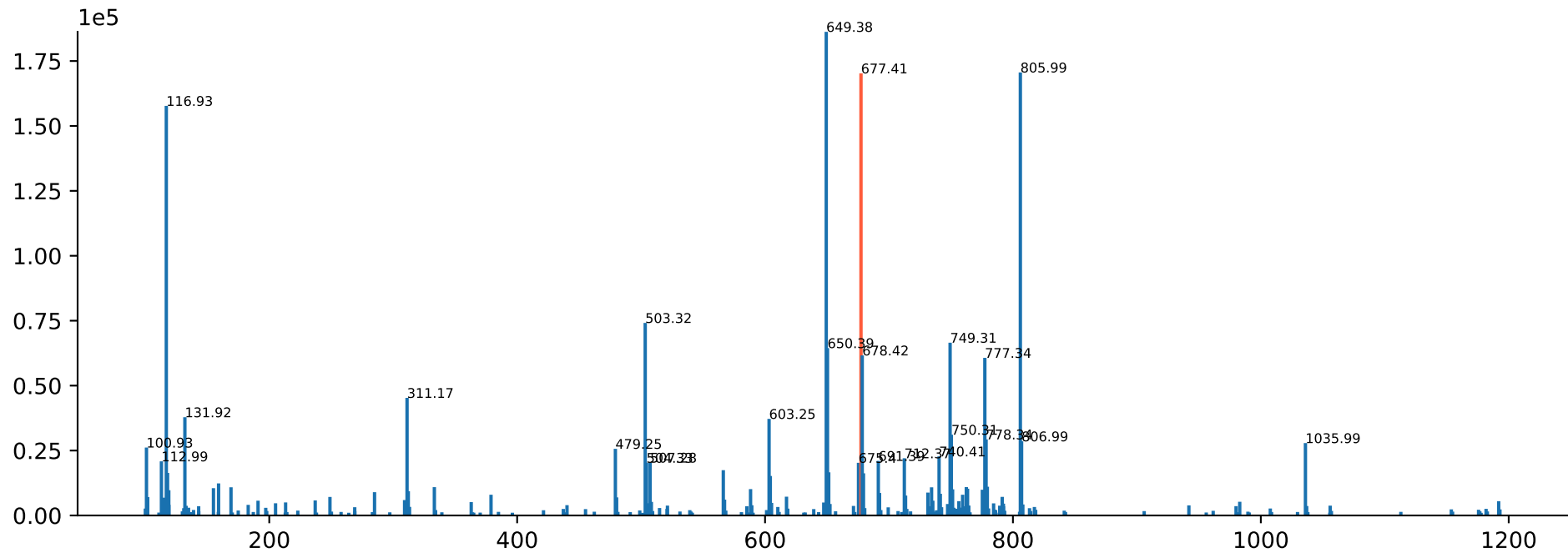

Supplement: Supplementary file 1 [file DataSheet1.zip › Supplementary files/Rhamnolipid Components in Fermentation Broth/Negpeak_group_000426.pdf]

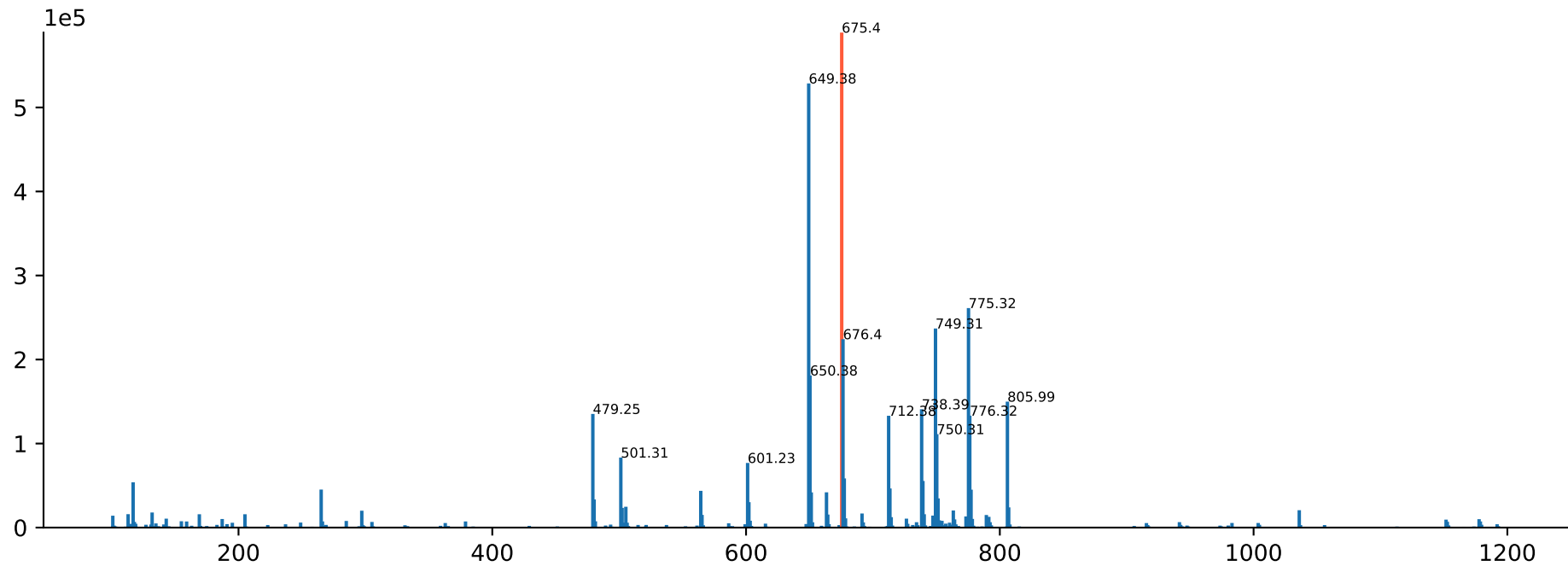

Supplement: Supplementary file 1 [file DataSheet1.zip › Supplementary files/Rhamnolipid Components in Fermentation Broth/Negpeak_group_000451.pdf]

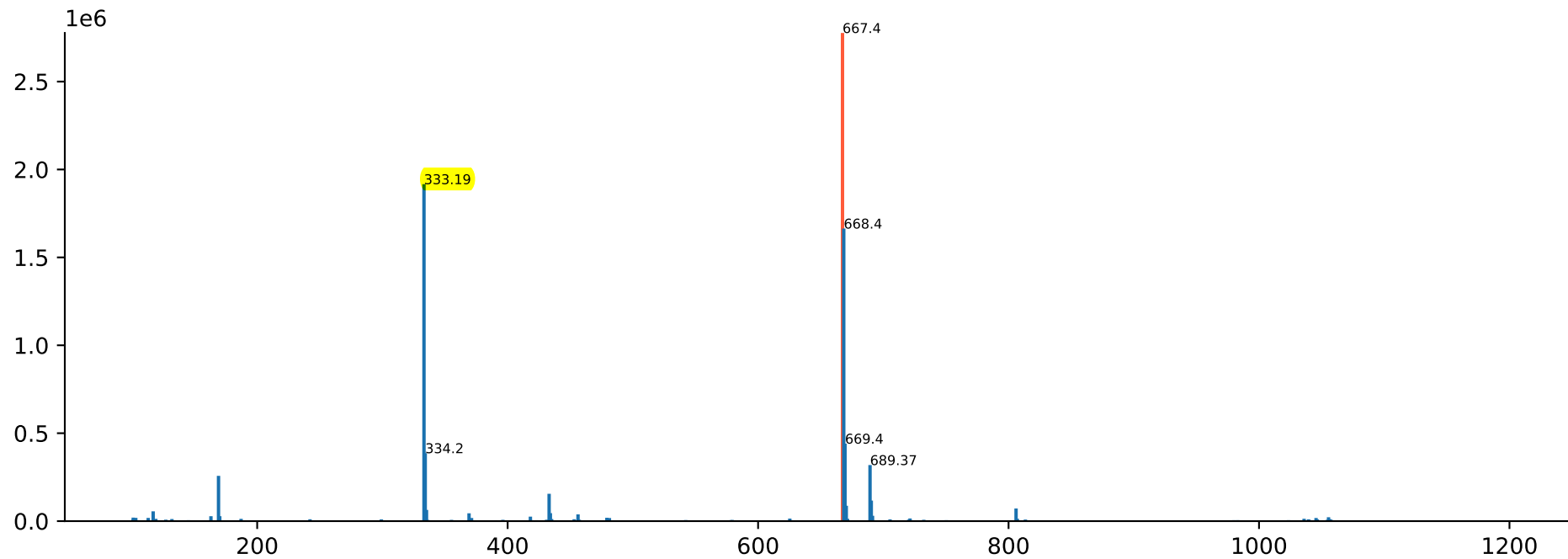

Supplement: Supplementary file 1 [file DataSheet1.zip › Supplementary files/Rhamnolipid Components in Fermentation Broth/Negpeak_group_000458.pdf]

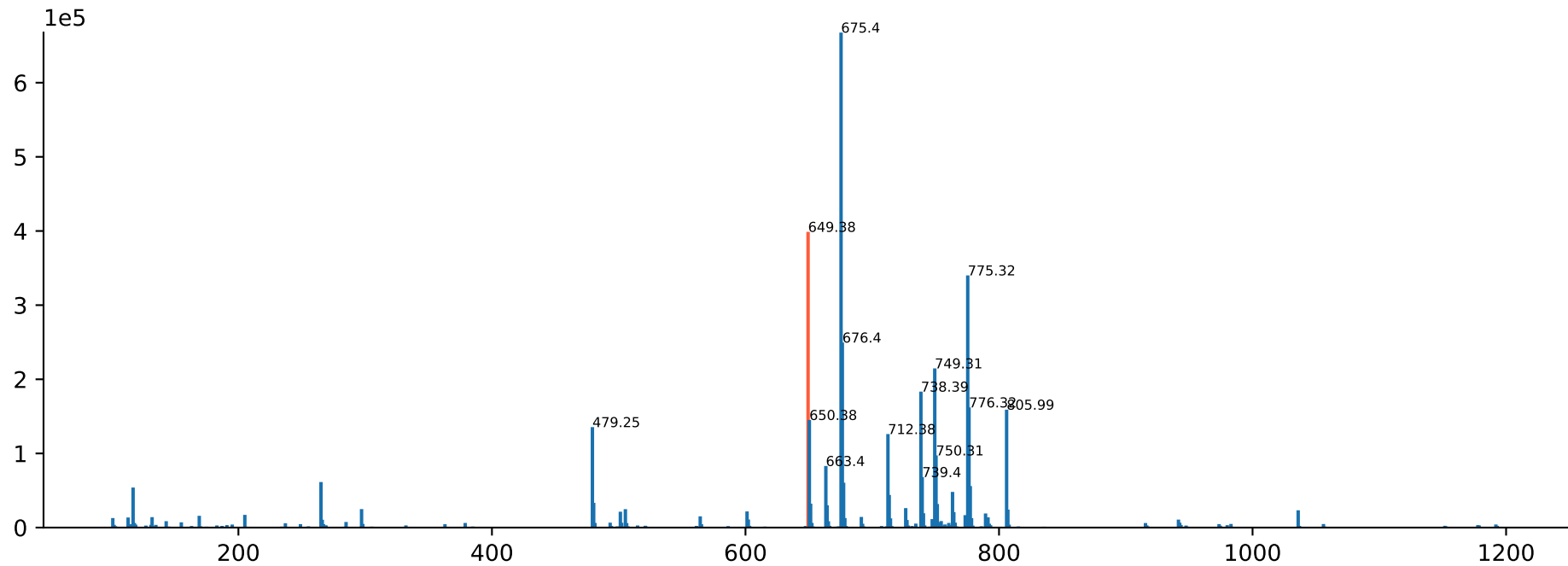

Supplement: Supplementary file 1 [file DataSheet1.zip › Supplementary files/Rhamnolipid Components in Fermentation Broth/Negpeak_group_000463.pdf]

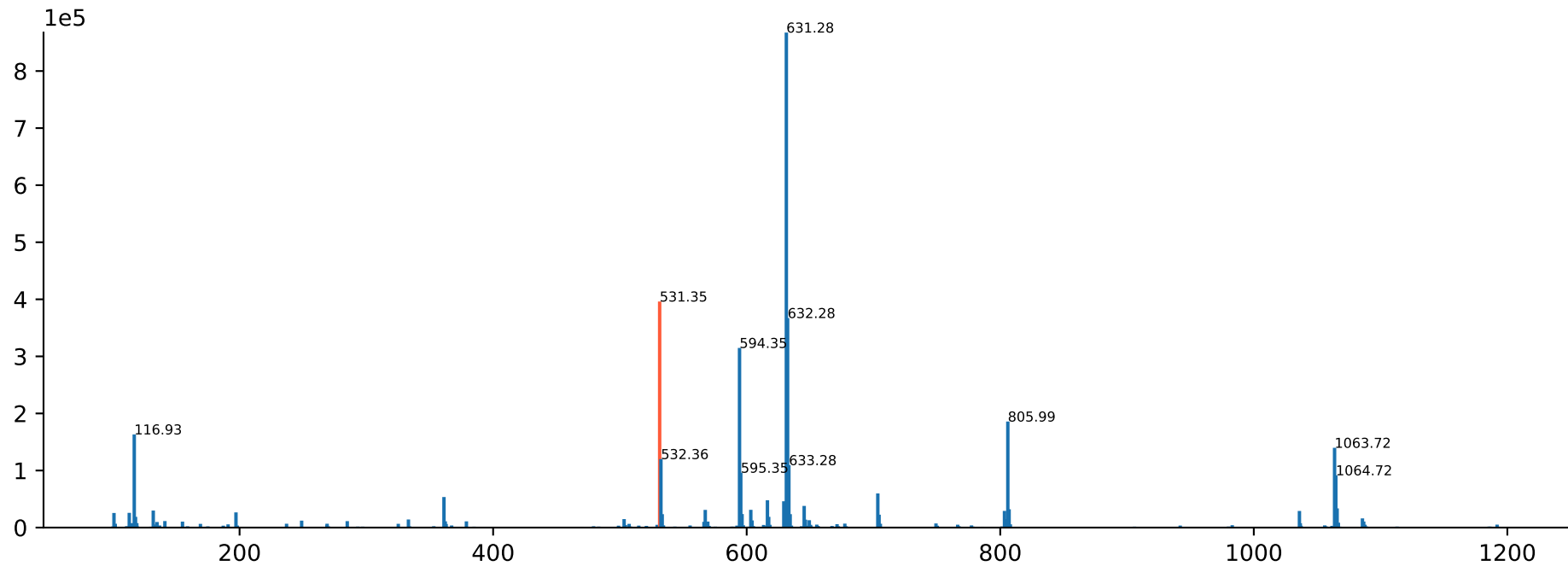

Supplement: Supplementary file 1 [file DataSheet1.zip › Supplementary files/Rhamnolipid Components in Fermentation Broth/Negpeak_group_000518.pdf]

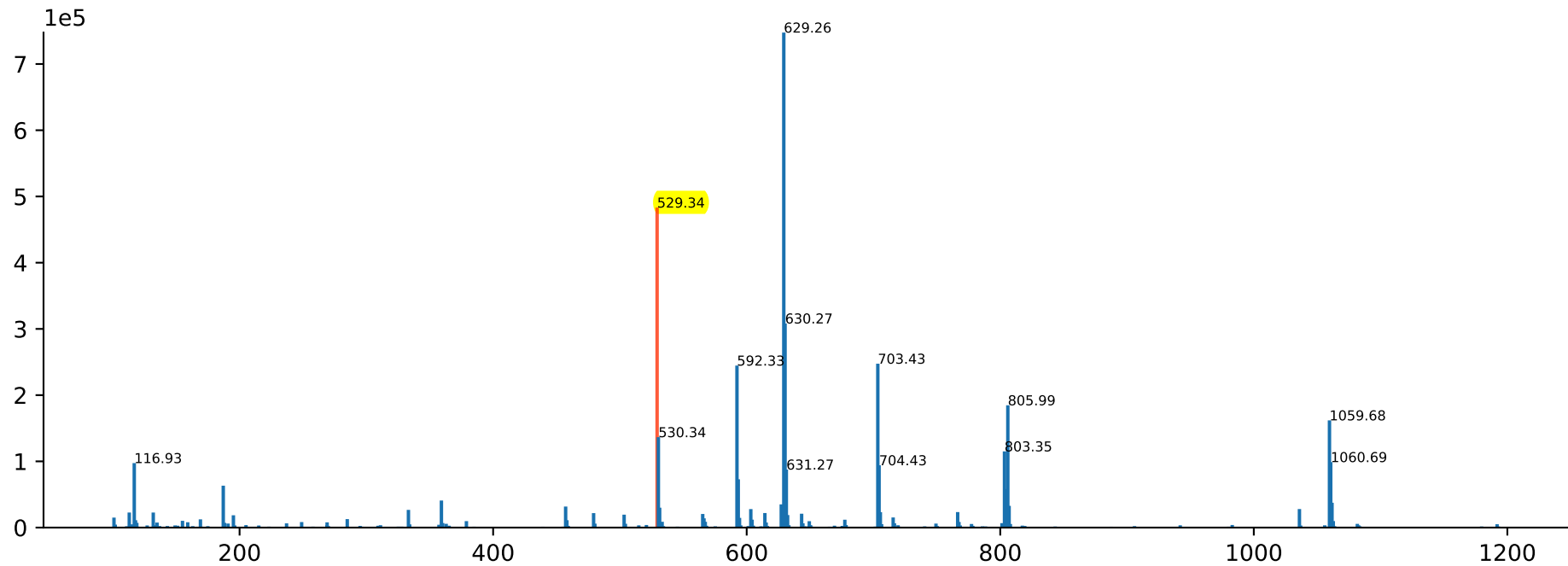

Supplement: Supplementary file 1 [file DataSheet1.zip › Supplementary files/Rhamnolipid Components in Fermentation Broth/Negpeak_group_000534.pdf]

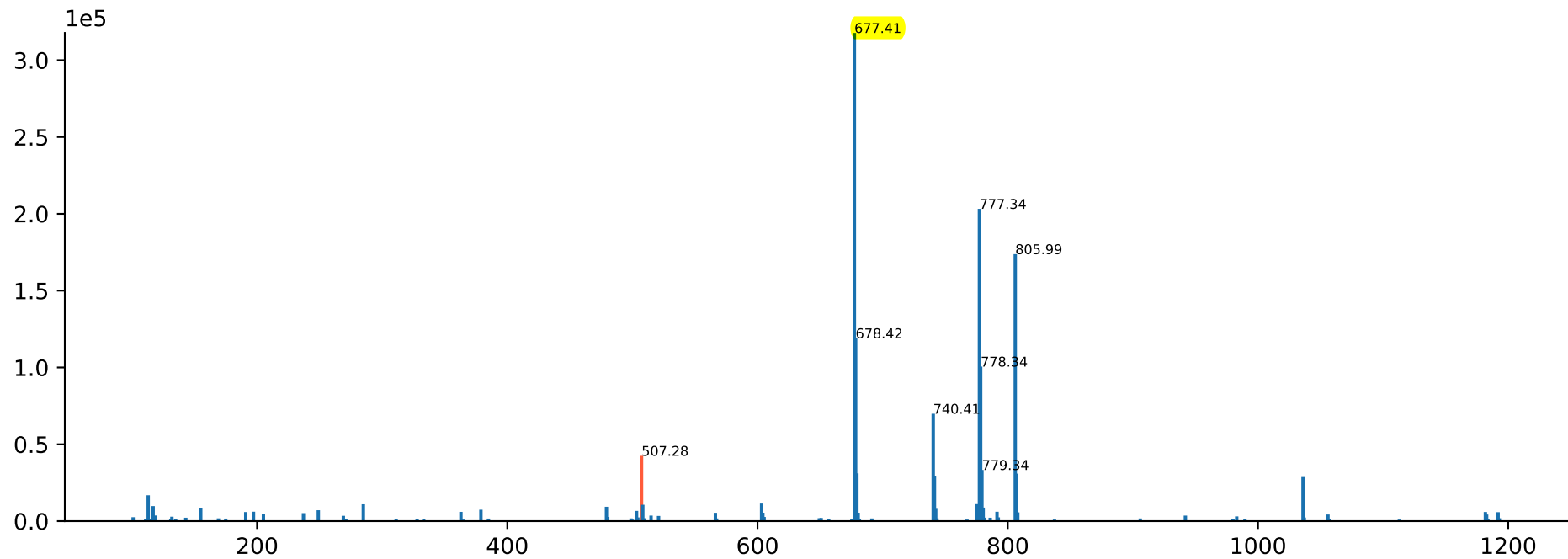

Supplement: Supplementary file 1 [file DataSheet1.zip › Supplementary files/Rhamnolipid Components in Fermentation Broth/Negpeak_group_000611.pdf]

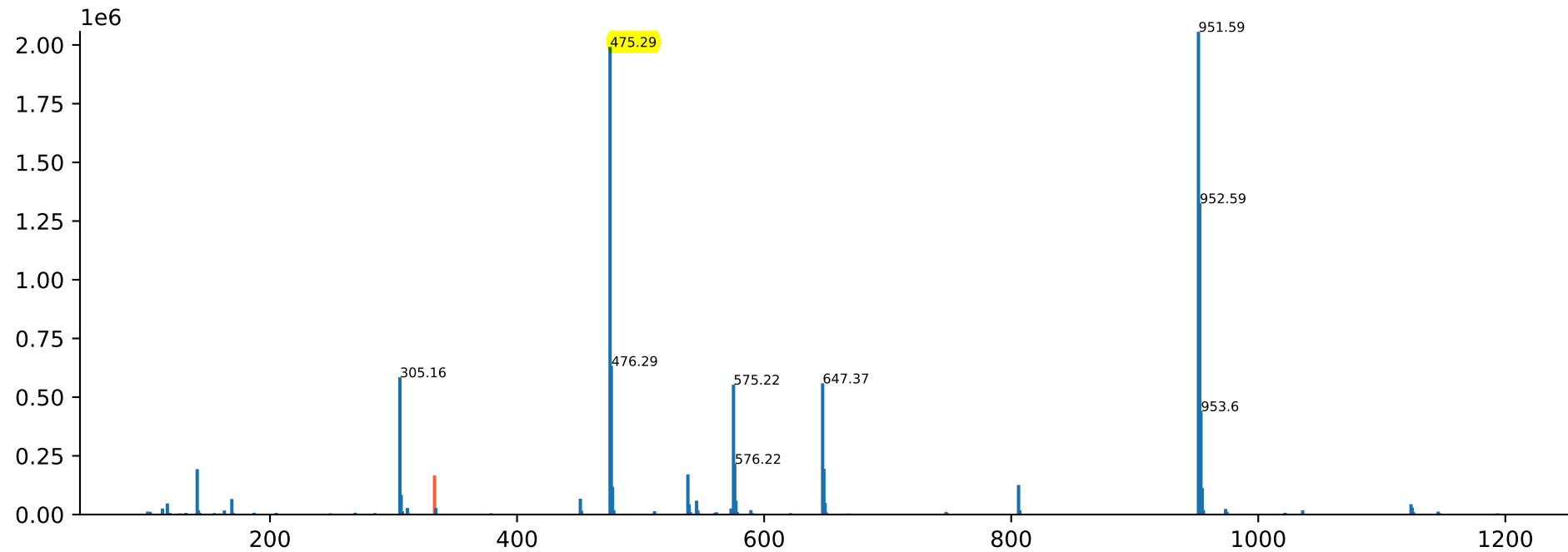

Supplement: Supplementary file 1 [file DataSheet1.zip › Supplementary files/Rhamnolipid Components in Fermentation Broth/Negpeak_group_000714.pdf]

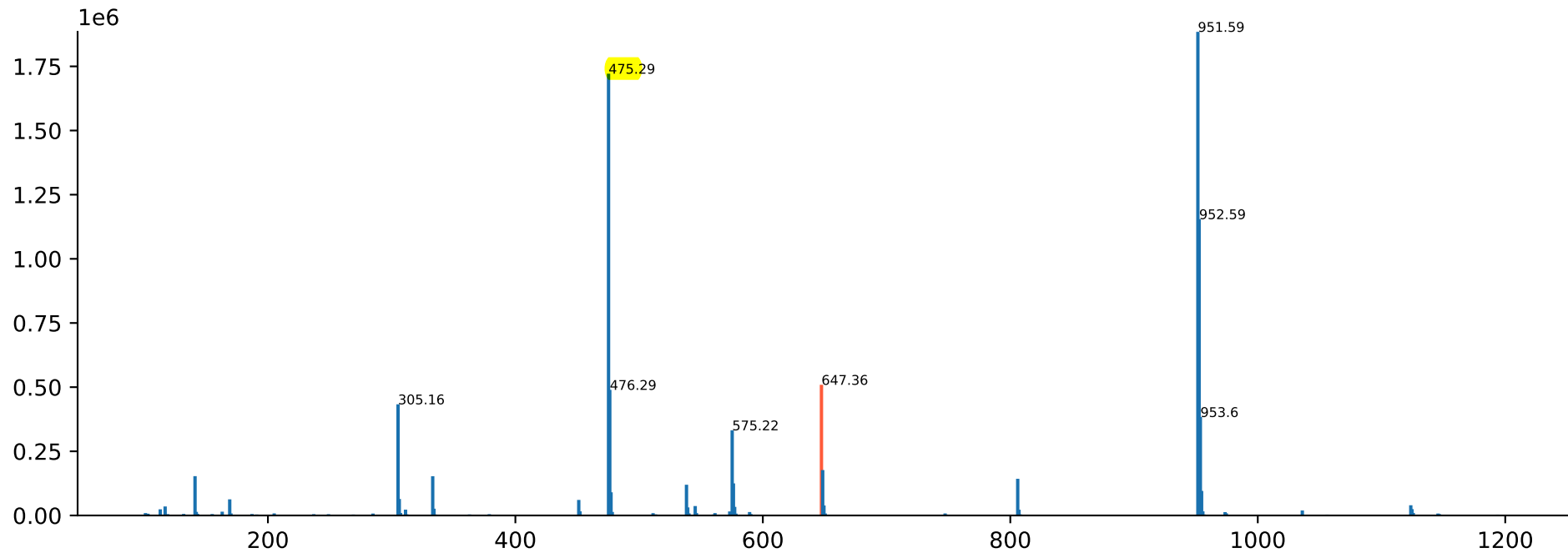

Supplement: Supplementary file 1 [file DataSheet1.zip › Supplementary files/Rhamnolipid Components in Fermentation Broth/Negpeak_group_000911.pdf]

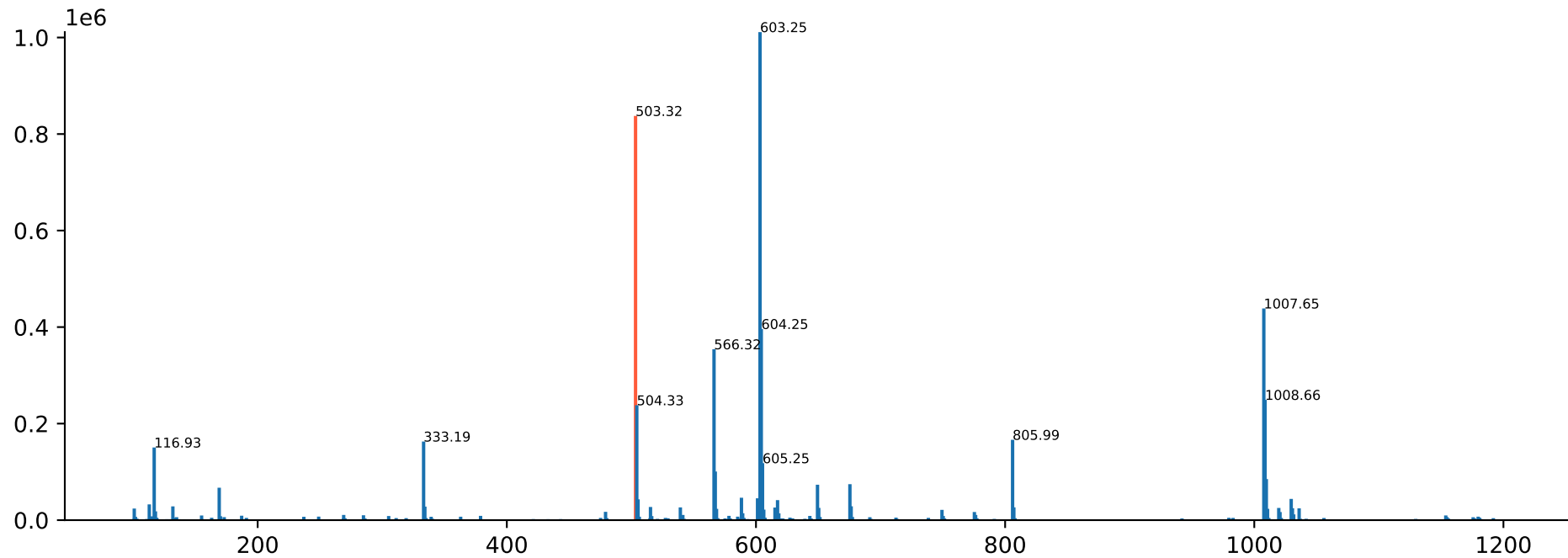

Supplement: Supplementary file 1 [file DataSheet1.zip › Supplementary files/Rhamnolipid Components in Fermentation Broth/Negpeak_group_001032.pdf]

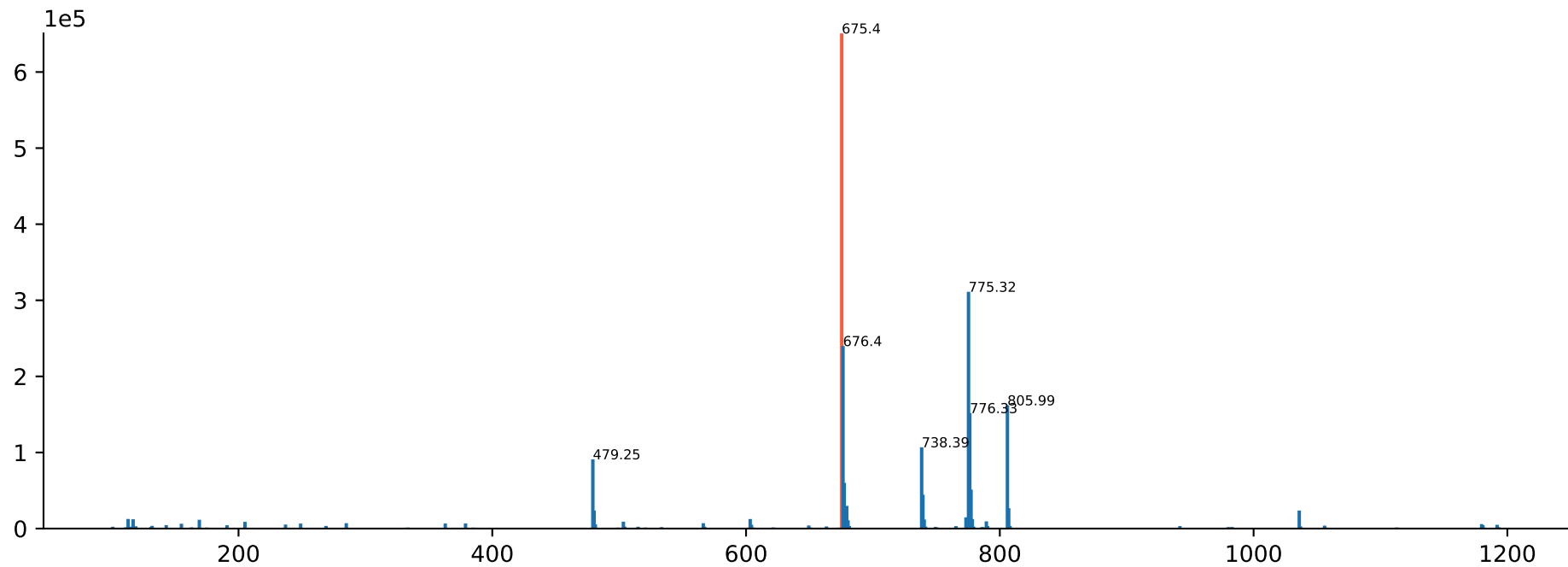

Supplement: Supplementary file 1 [file DataSheet1.zip › Supplementary files/Rhamnolipid Components in Fermentation Broth/Negpeak_group_001065.pdf]

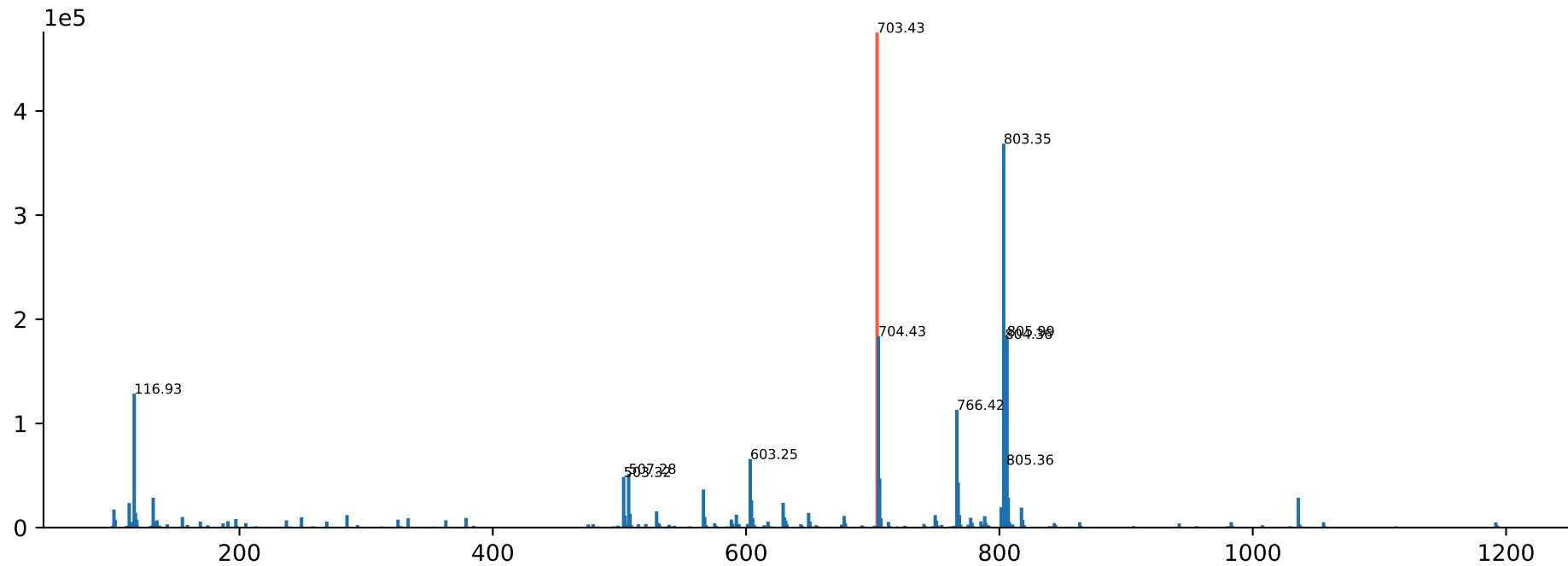

Supplement: Supplementary file 1 [file DataSheet1.zip › Supplementary files/Rhamnolipid Components in Fermentation Broth/Negpeak_group_001119.pdf]
